# Origin and diffusion of human Y chromosome haplogroup J1-M267

**DOI:** 10.1038/s41598-021-85883-2

**Published:** 2021-03-23

**Authors:** Hovhannes Sahakyan, Ashot Margaryan, Lauri Saag, Monika Karmin, Rodrigo Flores, Marc Haber, Alena Kushniarevich, Zaruhi Khachatryan, Ardeshir Bahmanimehr, Jüri Parik, Tatiana Karafet, Bayazit Yunusbayev, Tuuli Reisberg, Anu Solnik, Ene Metspalu, Anahit Hovhannisyan, Elza K. Khusnutdinova, Doron M. Behar, Mait Metspalu, Levon Yepiskoposyan, Siiri Rootsi, Richard Villems

**Affiliations:** 1 Estonian Biocentre, Institute of Genomics, University of Tartu, 51010 Tartu, Estonia; 2 Laboratory of Evolutionary Genomics, Institute of Molecular Biology of National Academy of Sciences of the Republic of Armenia, 0014 Yerevan, Armenia; 3 Lundbeck Foundation, Department of Biology, GeoGenetics Centre, University of Copenhagen, 1350 Copenhagen, Denmark; 4 Statistics and Bioinformatics Group, Institute of Fundamental Sciences, Massey University, Palmerston North, Manawatu 4442 New Zealand; 5 Institute of Cancer and Genomic Sciences, University of Birmingham, Birmingham, B15 2TT UK; 6 Department of Evolutionary Biology, Institute of Cell and Molecular Biology, University of Tartu, 51010 Tartu, Estonia; 7 ARL Division of Biotechnology, University of Arizona, Tucson, AZ 85721 USA; 8 Department of Genetics and Fundamental Medicine of Bashkir State University, Ufa, Bashkortostan Russia 450076; 9 Core Facility, Institute of Genomics, University of Tartu, 51010 Tartu, Estonia; 10 Institute of Biochemistry and Genetics of Ufa Federal Research Center of the Russian Academy of Sciences, Ufa, 450054 Russia; 11 Present Address: Thalassemia and Haemophilia Genetic PND Research Center, Dastgheib Hospital, Shiraz University of Medical Sciences, 71456-83769 Shiraz, Iran

**Keywords:** Evolution, Genetics

## Abstract

Human Y chromosome haplogroup J1-M267 is a common male lineage in West Asia. One high-frequency region—encompassing the Arabian Peninsula, southern Mesopotamia, and the southern Levant—resides ~ 2000 km away from the other one found in the Caucasus. The region between them, although has a lower frequency, nevertheless demonstrates high genetic diversity. Studies associate this haplogroup with the spread of farming from the Fertile Crescent to Europe, the spread of mobile pastoralism in the desert regions of the Arabian Peninsula, the history of the Jews, and the spread of Islam. Here, we study past human male demography in West Asia with 172 high-coverage whole Y chromosome sequences and 889 genotyped samples of haplogroup J1-M267. We show that this haplogroup evolved ~ 20,000 years ago somewhere in northwestern Iran, the Caucasus, the Armenian Highland, and northern Mesopotamia. The major branch—J1a1a1-P58—evolved during the early Holocene ~ 9500 years ago somewhere in the Arabian Peninsula, the Levant, and southern Mesopotamia. Haplogroup J1-M267 expanded during the Chalcolithic, the Bronze Age, and the Iron Age. Most probably, the spread of Afro-Asiatic languages, the spread of mobile pastoralism in the arid zones, or both of these events together explain the distribution of haplogroup J1-M267 we see today in the southern regions of West Asia.

## Introduction

After the major exodus from Africa, anatomically modern humans started their journey of colonizing Eurasia from West Asia^1,2^. Later this region harbored initial developments of other breakthroughs of the human past. The Neolithic demographic transition^3^, the origin of the first city-states and civilizations^4^, writing^5^, the use of metal-making^6^, and the emergence of Judaism, Christianity, and Islam^7^ are the most prominent ones.

Y chromosome haplogroup J-M304 represents the major male lineage in West Asia today^8–12^. The 12f2a^13^ deletion and single nucleotide polymorphic (SNP) biallelic markers M304^9^ and P209^14^ define and characterize this haplogroup. It splits off from haplogroup IJ-M429 at ~ 45 thousand years ago (kya), while the time to the most recent common ancestor (TMRCA) of haplogroup J-M304 lineages is ~ 33 kya^15,16^. Studies associate haplogroup J-M304 with the spread of farming from the Near East to Europe^11,17,18^. Around the time of the Neolithic demographic transition^3^, the genome-wide ancestry of West Asian populations was geographically structured into three groups^19–22^. Among them, haplogroup J-M304 is found in the Caucasus/Iranian and Anatolian hunter-gatherers and farmers, but not in the Levantine ones. Unfortunately, so far aDNA studies are missing from the Arabian Peninsula and Mesopotamia, where haplogroup J-M304 is frequent nowadays. This haplogroup splits into J1-M267 and J2-M172^9,11^. While haplogroup J2-M172 is associated more with agriculture in the northern latitudes of West Asia, haplogroup J1-M267 has been connected with the spread of the pastoral economies in the West Asian arid zones^23,24^.

**Figure 1:**
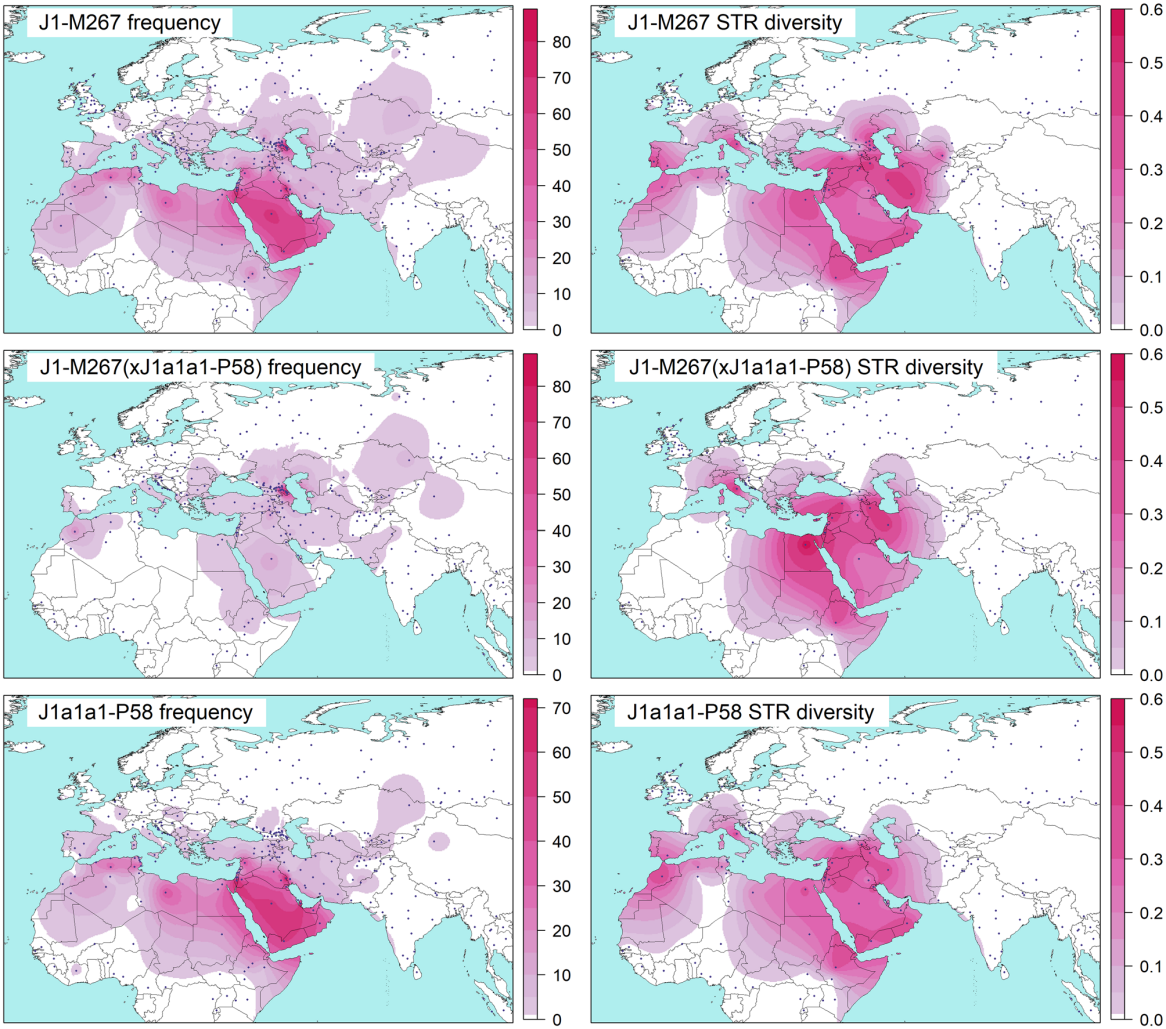
Spatial frequency and diversity distribution maps. Datasets for the frequency plots differ. Please note the difference in the scales. Data from Supplementary Table S7 are used to generate the frequency maps. The diversities were calculated with 8 short tandem repeats (STR) reported in Supplementary Table S8. Blue filled circles mark sampling locations. Maps were generated in RStudio software v1.2.5019 based on R software v3.6.1^77,78^. The base map was downloaded from http://tapiquen-sig.jimdo.com.

The distribution pattern of haplogroup J1-M267 is remarkable. It has two high-frequency regions—one in the Northeast Caucasus^10,25,26^ and another in the Arabian Peninsula, southern Mesopotamia, and the southern Levant^8,10,12,23,27^. The region between them has a lower frequency, but high genetic diversity^8–10,26,28^ (Fig. 1). A unique SNP marker—known as P58^14^ or Page8^23^—defines the major branch, which according to different classifications, is named as J1c3^14^, J1e^23^, J1b^15^, or J1a2a1a2 (https://isogg.org/tree/index.html v15.46). This branch is prevalent in the Arabian Peninsula, southern Iraq, and the Levant (Fig. 1).

Haplogroup J1-M267 probably evolved in the region encompassing northeastern Syria, southeastern Turkey, and northwestern Iran^23^ 9 to 24 kya^15,29–31^. The oldest human aDNA reported so far, belonging to this haplogroup, originates from an individual, who lived ~ 13.3 kya in the Caucasus during the Late Upper Paleolithic^21^. Previous studies have linked different past environmental, demographic, and cultural events to the current distribution of this haplogroup^8,10,12,23,24,27,29,32–38^. However, they suffer from limited phylogenetic resolution and sampling, and dating based on short tandem repeats (STR). Without proper reconstruction of phylogeny, it is problematic to address questions regarding the spatial and temporal aspects of the haplogroup. While whole Y chromosome resequencing studies have profoundly extended our understanding of this highly informative genetic locus^15,16,39–41^, each study includes only a limited number of haplogroup J1-M267 complete high-coverage sequences, if any at all (Supplementary Table S1).

In this study, we resequenced the male-specific region of 16 novel Y chromosomes of haplogroup J1-M267 with high-coverage (× 60–80). We have collected additional 156 from the literature (Supplementary Table S1). With the total of 172 high-coverage whole Y chromosomes, we reconstructed the detailed phylogeny and demographic history of haplogroup J1-M267. We infer the places of origin of haplogroups J1-M267 and J1a1a1-P58 in a statistically robust way using Bayesian continuous phylogeographic analysis. Further, we genotyped 39 phylogenetically informative SNP markers in 889 non-sequenced Y chromosomes of haplogroup J1-M267 from 37 populations. Lastly, we scanned the published ancient genomes of haplogroup J1-M267 representatives with 4292 phylogenetically informative SNP positions. We assess our results in the light of archaeological and linguistic findings and characterize the origin and diffusion of Y chromosome haplogroup J1-M267 and its major branch—haplogroup J1a1a1-P58.

## Results

**Figure 2:**
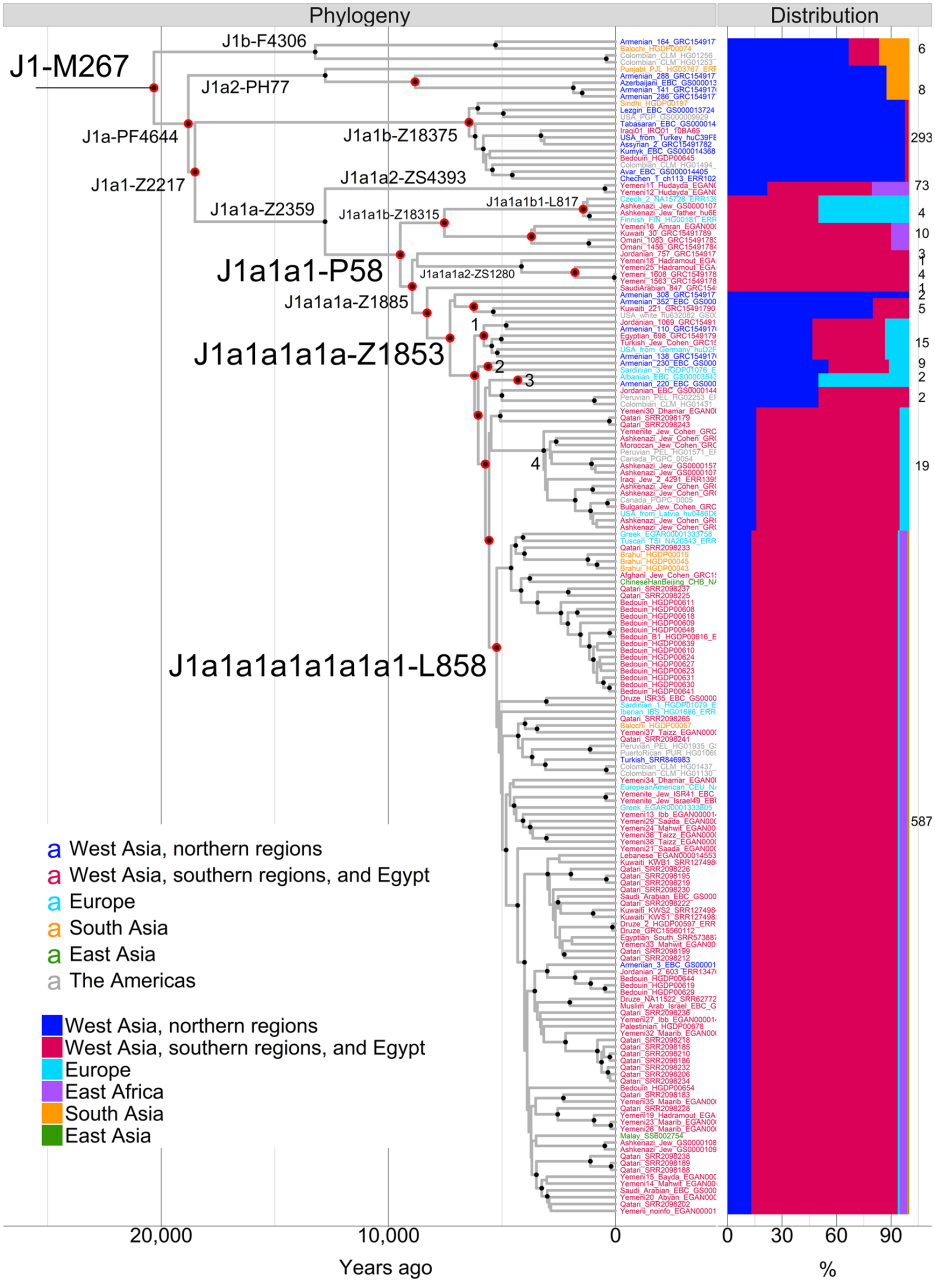
Bayesian time-scaled maximum clade credibility tree of haplogroup J1-M267 and the distribution of the genotyped branches. The phylogeny is reconstructed with Beast v1.10.4 software^82^. Black filled circles mark the nodes with posterior probability ≥ 0.95. Red filled circles mark the nodes genotyped in the genotyping dataset. Tip labels are color coded according to geography. The frequencies of the branches are calculated out of 158 sequenced and 889 genotyped Eurasian and African samples belonging to that branch. The overall numbers are denoted at the rightmost side of the plot. The numbers written at the tree nodes refer to the following branches: 1—J1a1a1a1a1b-Z18297, 2—J1a1a1a1a1a2-ZS2524, 3—J1a1a1a1a1a1b-B2069, 4—J1a1a1a1a1a1a2-B877.

We reconstructed the Y chromosome haplogroup J1-M267 phylogeny with 172 high-coverage sequences of modern humans using 4292 high-quality SNPs (Fig. 2, Supplementary Fig. S1, Supplementary Table S2). Recurrent SNPs account for ~ 0.26%, which is comparable with other studies^15,39^.

We estimated the TMRCA of haplogroup J1-M267 to be ~ 20.3 kya with 95% highest posterior density (HPD) interval of 16.3–24.4 kya (Fig. 2, Table 1, Supplementary File S1). The estimate overlaps with those suggested previously^15,23^ and with the Last Glacial Maximum (LGM) (26.5–19.0 kya)^42^. The Y chromosome mutation rate derived from our analysis is equivalent to 6.95e^−10^ mutations/bp/year (95% HPD = 5.56e^−10^–8.51e^−10^), which is comparable with earlier estimates^15,43,44^. The coefficient of the mutation rate variation equals to 0.0932 (95% HPD = 0.013–0.161). This shows that the phylogeny of haplogroup J1-M267 evolves in a clock-like manner and has similar rates of substitutions on different branches.

**Table 1:** Age estimates of haplogroup J1-M267 and its branches.

Haplogroup	Age	95% HPD intervals	Number of genotyped markers	Haplogroup in^15^	Haplogroup in ISOGG v15.46
J1-M267	20.3	16.3–24.4	2	J1	J1
J1a-PF4644	18.8	15.1–22.4	2	J1	J1a
J1b-F4306	13.4	10.2–16.6	0	_	J1b
J1a1-Z2217	18.5	14.9–22.1	1	J1a’b	J1a2~
J1a2-PH77	12.8	9.9–15.6	0	J1c	J1a3~
J1a1a-Z2359	12.8	9.9–15.7	0	_	J1a2a~
J1a1b-Z18375	6.5	5.0–8.0	2	J1a	J1a2b
J1a1a1-P58	9.5	7.4–11.7	1	J1b	J1a2a1a2
J1a1a2-ZS4393	0.5	0.1–1.0	0	_	J1a2a1a1~
J1a1a1b-Z18315	7.6	5.7–9.4	2	J1b7	J1a2a1a2c~
J1a1a1b1-L817	1.5	0.9–2.1	2	J1b7	J1a2a1a2c1
J1a1a1a2-ZS1280	4.2	2.9–5.6	0	_	J1a2a1a2d1~
J1a1a1a1a-Z1853	7.3	5.7–9.0	5	J1b1′5	J1a2a1a2d2b~
J1a1a1a1a1b-Z18297	5.8	4.5–7.2	1	_	J1a2a1a2d2b2a~
J1a1a1a1a1a2-ZS2524	5.7	4.4–7.0	1	J1b5	J1a2a1a2d2b2b1~
J1a1a1a1a1a1b-B2069	4.3	3.1–5.7	1	J1b3	_
J1a1a1a1a1a1a1-L858	5.3	4.1–6.5	3	J1b1*	J1a2a1a2d2b2b2c4~
J1a1a1a1a1a1a2-B877	3.2	2.4–4.0	0	J1b2*	J1a2a1a2d2b2b2c2a~

Coalescence times and 95% HPD intervals were estimated with Beast v1.10.4^82^ and are expressed in thousand years ago. For comparison purposes, the branch names in other Y chromosome trees are provided also. Only the branches discussed in the main text are listed. Underscore means the branch is missing.

*The branch names were later updated^29^.

Haplogroup J1-M267 is defined by 103 SNP markers in our reconstructed phylogeny (Supplementary Fig. S1, Supplementary Table S2). The J1b-F4306 and J1a2-PH77 branches split off at the very beginning (Fig. 2). These are rare lineages, distributed mostly among ancient and modern populations from the Caucasus, the Armenian Highland, Iran, and Pakistan (Fig. 2, Supplementary Fig. S1, Supplementary Tables S3, S4). In addition to a similar geographical distribution, the J1b-F4306 and J1a2-PH77 branches also have similar TMRCA, ~ 13.2 kya (95% HPD = 10.2–16.6 kya) and ~ 12.8 kya (95% HPD = 9.9–15.6 kya), respectively (Table 1). Interestingly, the ~ 13 thousand-year-old hunter-gatherer^21^ from the South Caucasus belongs to J1b-F4306.

The J1a1b-Z18375 branch has a geographic distribution substantially overlapping with that of the J1b-F4306 and J1a2-PH77 branches. The J1a1b-Z18375 branch is found overwhelmingly among ancient and modern populations from the Caucasus, the Armenian Highland, Iran, and Anatolia (Fig. 2, Supplementary Fig. S1, Supplementary Tables S3, S4). This branch constitutes the aforementioned peak of haplogroup J1-M267 frequency in the Northeast Caucasus. All members of this branch, genotyped for DYS388 STR, have alleles with 14 or fewer repeats. These “short” DYS388 alleles in haplogroup J1-M267 were found among populations living in the Caucasus and neighboring regions^9,26^. Unlike the other two branches mentioned above, J1a1b-Z18375 coalesces later ~ 6.5 kya (95% HPD = 5.0–8.0 kya) (Table 1).

The J1a1a-Z2359 branch divides into two branches—the well-known major J1a1a1-P58 and J1a1a2-ZS4393. J1a1a-Z2359 is found in the Chalcolithic northern Levant close to Anatolia and among the Copper Age/Eneolithic and Early Bronze Age individuals in Central Asia, adjacent to the present-day northern border with Iran^45,46^. All these individuals share genome-wide ancestry with either Anatolian or Iranian Neolithic/Mesolithic populations. The early presence of the J1a1a-Z2359 branch adjacent to present-day northern Iran suggests the origin of this branch in the northern West Asian regions. The J1a1a2-ZS4393 branch consists of two recently diverged samples from Yemen^47^. Our phylogeographic survey has identified specimens likely connected to this branch. These specimens occur over a wide geographic area encompassing West Asia and East Africa (Supplementary Table S4). In East Africa, 40% of haplogroup J1-M267 lineages belong to this group. Alternatively, these specimens can be a paragroup. Further sequencing of the Y chromosomes of these samples will shed more light on the demographic history of the J1a1a2-ZS4393 branch and the J1a1a-Z2359 branch in general.

J1a1a1-P58 represents the major branch of haplogroup J1-M267 (Figs. 1, 2, Supplementary Fig. S1, Supplementary File S1). The frequency culminates in the Arabian Peninsula, southern Mesopotamia, and the southern Levant. Moderate frequencies occur in the northern parts of West Asia, North Africa, and East Africa. Lower frequencies occur in Europe, Central Asia, and South Asia. It is defined by 23 SNPs and coalesces 9.5 kya (95% HPD = 7.4–11.7 kya) (Table 1, Supplementary File S1).

Early splitting of the J1a1a1-P58 branch results in the minor branches and singleton lineages distributed overwhelmingly among the modern populations of the Arabian Peninsula, the Levant, southern Mesopotamia, and East Africa (Supplementary Table S4). These are J1a1a1b-Z18315, J1a1a1a2-ZS1280, J1a1a1a3-B2146 and J1a1a1a1b-B2062. In the northern parts of West Asia, they are virtually absent. The ancient distribution of these branches mirrors the current one (Supplementary Table S3). A notable exception is J1a1a1b1-L817, a sub-branch of J1a1a1b-Z18315; while its modern members are found among the Ashkenazi Jews and central and northeastern Europeans, it is also found in a 300–500 CE individual from Rome^48^. The TMRCA of J1a1a1b1-L817—1.5 kya (95% HPD = 0.9–2.1 kya) (Table 1)—corresponds to the age of other Ashkenazi Jewish Y chromosome founders^29^. The distribution of these branches in the southern regions of West Asia suggests the origin of the J1a1a1-P58 branch there.

A sub-branch of J1a1a1-P58—J1a1a1a1a-Z1853—coalesces ~ 7.3 kya (95% HPD = 5.7–9.0 kya) (Table 1, Supplementary File S1). This branch retains an important phylogeographic mark as the branch to which most of the northern West Asian J1a1a1-P58 lineages belong (Fig. 2, Supplementary Fig. S1). Nevertheless, the J1a1a1a1a-Z1853 branch occurs primarily in the southern regions of West Asia. Most of the ancient members are found in the Levant and Egypt (Supplementary Table S3). Among them is the oldest known aDNA, belonging to haplogroup J1a1a1-P58^46^ (Supplementary Table S3). Minor branches of the J1a1a1a1a-Z1853 contain samples from Europe as well. These are J1a1a1a1a1b-Z18297, J1a1a1a1a1a2-ZS2524 and J1a1a1a1a1a1b-B2069 (Fig. 2, Supplementary Fig. S1), coalescing ~ 4–6 kya (Table 1, Supplementary File S1). Another sub-branch—J1a1a1a1a1a1a2-B877—is specific to the Jewish Cohens (Fig. 2, Supplementary Fig. S1). Its TMRCA of ~ 3.2 kya (95% HPD = 2.4–4.0 kya) overlaps with the previous estimate^29^ (Table 1, Supplementary File S1). It exceeds the TMRCA of J1a1a1b1-L817, another Jewish lineage in haplogroup J1-M267. Intriguingly, an ancient member of the J1a1a1a1a1a1a2-B877 branch is found again in Rome, this time in the Imperial period (27 BCE—300 CE), which is characterized by an increase in genome-wide ancestry from the eastern Mediterranean region^48^.

The J1a1a1a1a1a1a1-L858 branch—coalescing ~ 5.3 kya (95% HPD = 4.1–6.5 kya)—is the biggest sub-branch not only of J1a1a1a1a-Z1853, but also of the whole J1-M267 (Fig. 2, Supplementary Table S4). More than half (~ 56%) of the modern members of haplogroup J1-M267 belong to this branch. The proportion becomes even larger (~ 88%) if we consider only haplogroup J1a1a1-P58 chromosomes. The great majority (~ 80%) of members of the J1a1a1a1a1a1a1-L858 branch are distributed in the Arabian Peninsula, the Levant, southern Mesopotamia, and Egypt. Another ~ 4% are dispersed in East Africa among the Afro-Asiatic-speaking populations of Ethiopia. It splits extensively forming a large number of downstream branches (Fig. 2, Supplementary Fig. S1, Supplementary File S1). Many branches exclusively include members of the Arabic-speaking populations. In contrast, a small number of minor branches contain only members of non-Arabic-speaking populations. These branches bear fewer than four individuals per branch. Other non-Arabic-speaking individuals are scattered as singleton lineages in the Arabic-specific branches. Specimens of two different Arabic-speaking populations coalesce mostly between ~ 2 and ~ 5 kya as do many population-specific branches (Supplementary Fig. S1, Supplementary File S1). Ancient specimens of J1a1a1a1a1a1a1-L858 are found mostly in the Levant (Supplementary Table S3).

The phylogeny of haplogroup J1-M267 is described in detail in Supplementary Note.

### Origin of haplogroups J1-M267 and J1a1a1-P58

**Figure 3:**
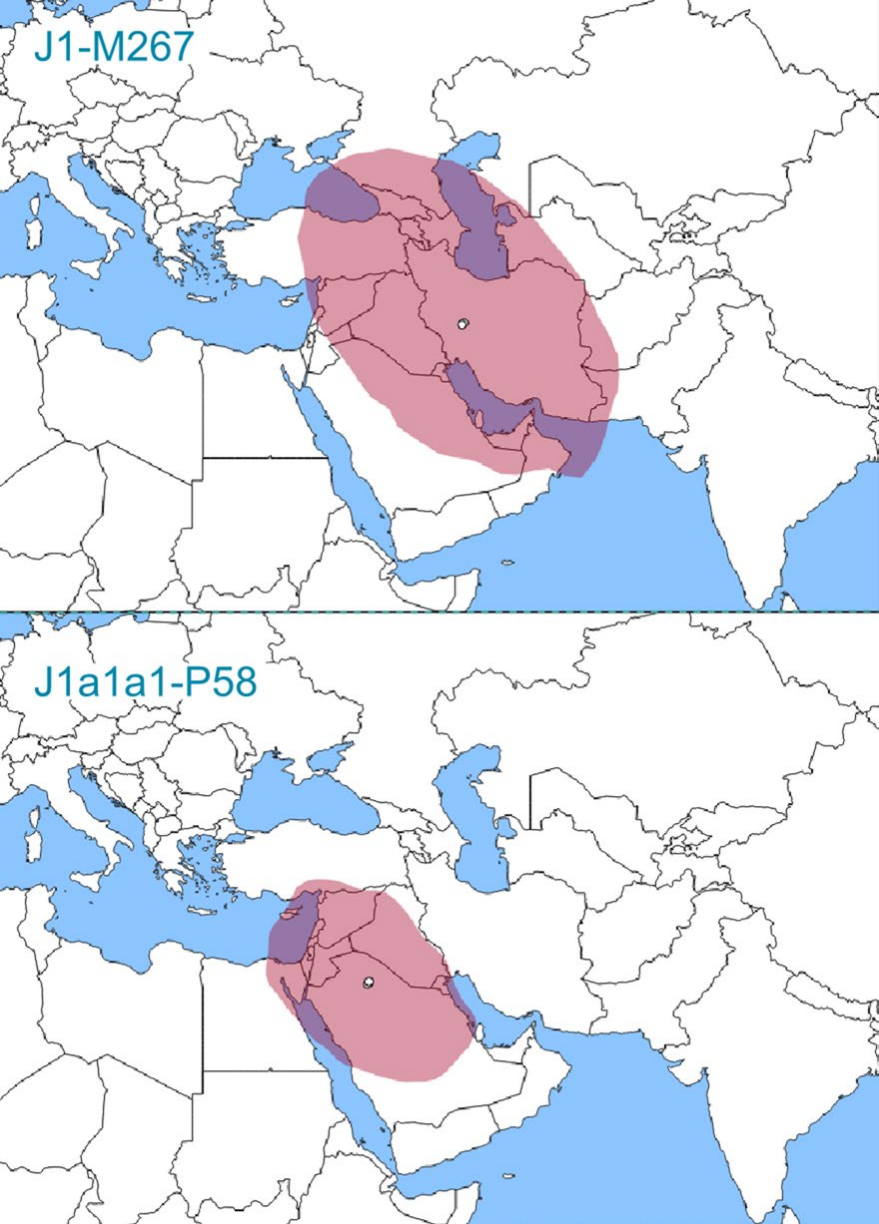
Inferred root locations of haplogroups J1-M267 and J1a1a1-P58. Shaded in pink is the 80% HPD area of the root locations inferred by Bayesian continuous phylogeographic analysis in Beast v1.10.4 software^82^. Open circles show median estimates. Maps were generated in spreaD3 software (v0.9.7.1rc)^90^. The base map was downloaded from https://github.com/johan/world.geo.json/blob/master/countries.geo.json.

We infer the area of origin of haplogroups J1-M267 and J1a1a1-P58 with Bayesian continuous phylogeographic analysis. This statistically robust method considers the coalescent, phylogenetic, molecular clock, location, and other uncertainties within a single framework^49,50^. Another important advantage of this analysis is that it is absolutely data-driven and doesn’t require a prior definition of geographic groupings. We estimate diffusion rates for haplogroups J1-M267 and J1a1a1-P58 as 0.3134 (95% HPD = 0.2446–0.3828) and 0.361 (95% HPD = 0.2831–0.4494) kilometers/year, respectively. The inferred 80% HPD area of the haplogroup J1-M267 root’s locations covers Iran, the Caucasus, the Armenian Highland, Mesopotamia, the northern Levant, and the northern and the eastern Arabian Peninsula (Fig. 3). For haplogroup J1a1a1-P58, the inferred 80% HPD area of the root locations covers the Arabian Peninsula, the Levant, northeastern Egypt, Mesopotamia, Cyprus, and a small area of the coastal region of Anatolia. These results corroborate the extensive phylogeographic analysis with a broader sample of genotyping (Supplementary Table S4).

### The demographic history of haplogroup J1-M267

**Figure 4:**
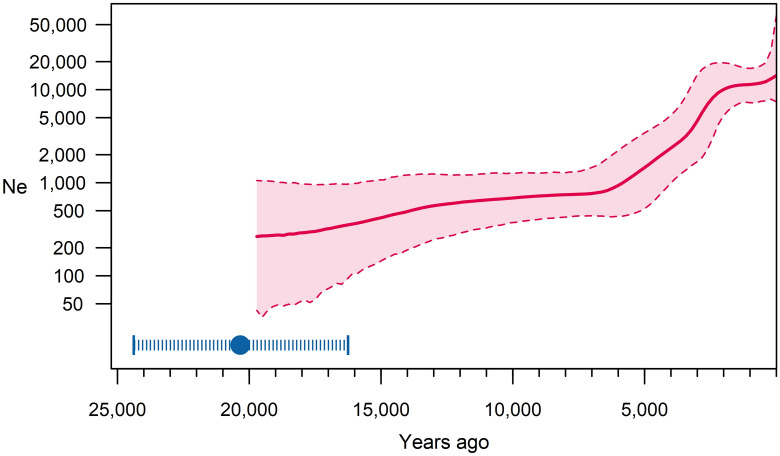
Bayesian skyline plot of haplogroup J1-M267. The solid line is the median estimate, while the dashed lines show the 95% HPD limits. Mean (filled circle) and 95% HPD intervals (pipes) for haplogroup J1-M267 coalescence time is provided in the figure. *N*_*e*_ effective population size.

We reconstructed the demographic history of haplogroup J1-M267 with Bayesian skyline analysis of its effective population size (Ne). Ne increases slightly, if at all, during the time from its origin at the late LGM until ~ 6 kya (Fig. 4). Although the median and 95% HPD intervals do increase slightly, the Ne value of the lowest 95% HPD bound remains lower than that of the highest 95% HPD bound at the end of this initial period. Therefore, we consider this change as negligible. This time interval of basically constant Ne includes also the early Holocene when the Neolithic demographic transition occurred^3^. It suggests that the ancestral population of haplogroup J1-M267 was not impacted by the putative population expansion during the Neolithic. After ~ 6 kya, Ne of haplogroup J1-M267 grows intensively until ~ 2 kya. Unlike the previous period, here the lowest 95% HPD bound of Ne overcomes its highest 95% HPD bound at ~ 6 kya. Hence, we have evidence of a substantial increase of haplogroup J1-M267 Ne between 6 and 2 kya. Afterwards, during the last ~ 1.5 ky Ne increases slightly, which, again, we consider as negligible. Thus, the major expansion of haplogroup J1-M267 took place during the Chalcolithic, the Bronze Age, and the Iron Age. Before and after this time, Ne remained essentially constant.

## Discussion

In this study, we reconstructed the Y chromosome haplogroup J1-M267 phylogenetic tree (Fig. 2, Supplementary Fig. S1, Supplementary File S1) with a large number of high-coverage whole Y chromosome sequences. We conducted phylogeographic and demographic analyses with the Bayesian MCMC approach (Figs. 3, 4). Using this refined tree, we explicitly follow the divergence pattern in haplogroup J1-M267. It starts ~ 20 kya, which fits the late LGM timeframe (Table 1, Fig. 2, Supplementary File S1) and corresponds to previously reported estimates^15,23^. The origin at or immediately after the last stage of LGM mirrors other uniparental West Asian lineages, such as Y chromosome haplogroups G2a-P15, J2a-M410, J2b-M12^15^, and mitochondrial DNA haplogroups U7^51^ and HV^52^, among others. The loss of diversity in these haplogroups, including J1-M267, might be from the severe glacial conditions, which left only a few founders.

Our Bayesian continuous phylogeographic analysis suggests the origin of haplogroup J1-M267 in West Asia, confirming previous assumptions^8,23,28^. Most of the deeply diverged lineages belong to the populations from the northern latitudes of the region, in line with the finding of the late Pleistocene hunter-gatherer^21^ from the South Caucasus. Our analyses provide better statistical confidence for the origin of haplogroup J1-M267 in the area encompassing the Caucasus, the Armenian Highland, and the Zagros Mountains. Ancient populations living in the Caucasus and the Zagros Mountains also shared genome-wide ancestry, differing from that of the Levantine and Anatolian populations during the late Pleistocene and early Holocene^21,22^. Therefore, the Y chromosome haplogroup J1-M267 and genome-wide ancestry support similar conclusions about human demography in the region at this time.

At least three branches of the haplogroup J1-M267 diversified ~ 13 kya (Fig. 2, Table 1). These are J1b-F4306, J1a2-PH77, and J1a1a-Z2359. During these cold and harsh climatic conditions—known as the Younger Dryas^53^ (11.7–12.9 kya)^54^—the mountainous areas in northern West Asia likely ensured enough resources for the survival of the populations among which these lineages evolved.

The frequency of modern variation and the phylogeny of the J1a1b-Z18375 branch suggest an origin in the Caucasus or the immediate vicinity. Compared to other Caucasian lineages (J1b-F4306 and J1a2-PH77), this branch diversified later: in the Chalcolithic or the Bronze Age ~ 6.5 kya (95% HPD = 5.0–8.0 kya). Interestingly, an ancient individual from the Caucasus^55^ belonging to J1a1b-Z18375 is found in the assemblage of the Bronze Age Kura-Araxes cultural tradition^56^. This cultural tradition probably originated in the Caucasus and may explain the radiation of this branch. This finding is consistent with the autosomal DNA analysis of the Anatolian Bronze Age individuals who trace ~ 32% of their ancestry ultimately from the Caucasus hunter-gatherers/Iranian farmers^57^. The presence of the ancient Levantine J1a1b-Z18375 individuals^58^ and the sub-branch composed by an Assyrian, an Iraqi, and an individual with Turkish ancestry, coalescing ~ 3.3 kya (95% HPD = 2.4–4.3 kya), may be explained by the suggested connection of the Hurro-Urartian languages with the eastern Caucasian languages within the so-called Alarodian language family^59^. More samples are necessary to better understand the demographic processes that shaped the distribution of the J1a1b-Z18375 branch.

Haplogroup J1a1a1-P58 diverged starting from ~ 9.5 kya (95% HPD = 7.4–11.7), i.e. during the early Holocene. This time corresponds to the origin of farming in West Asia. Nevertheless, the population size of haplogroup J1-M267 remained small during the early Holocene, contrary to the conclusions suggested previously^23,37^. Phylogeographic analysis reveals that the initial divergence results in lineages mostly present in the Arabian Peninsula, the southern Levant, and southern Mesopotamia. In contrast, all, but one northern West Asian representatives of this branch belong to the lineage coalescing only ~ 7.3 kya (95% HPD = 5.7–9.0). On average, this postdates the TMRCA of the entire haplogroup J1a1a1-P58 by ~ 2 ky. Bayesian phylogeographic analysis, genotypes of additional samples as well as the prevalence of haplogroup J1a1a1-P58 support its origin in the southern regions of West Asia. Our conclusions contradict the earlier suggestions based on the high diversity of Y chromosome STRs in the populations of the Zagros and Taurus Mountains and northern Mesopotamia^8,23^. However, the high STR diversity may be better explained by the different coalescence patterns of haplogroup J1a1a1-P58 in the northern versus the southern regions of West Asia. A larger number of samples—coalescing, on average, to more recent times in the southern regions—may shift the modal haplotype from the root haplotype and bias their STR-based TMRCA estimates. Moreover, earlier studies noticed high STR-based TMRCAs in the southern populations of the Arabian Peninsula^23^. Other issues with STR-based TMRCA estimates relate to saturation, homoplasy, non-linear accumulation of molecular changes, and large differences between suggested rates^15,39,60^. We conclude that haplogroup J1a1a1-P58 started to diverge most probably in a region encompassing the Arabian Peninsula, the southern Levant, and southern Mesopotamia. The small-scale migration of people from northern West Asia to these regions among whom J1a1a1-P58 evolved, obviously occurred between the origins of J1a1a-Z2359 and J1a1a1-P58. The most conservative estimate is 7.4–15.7 kya.

Haplogroup J1a1a1-P58 in ancient populations was found only in the Bronze Age Levant^22,46,61^. It is worth to note that the aDNA studies lack samples from the Arabian Peninsula and Mesopotamia, leaving us with only a few male samples of the appropriate age from the southern regions of West Asia. The initial low absolute number of people bearing haplogroup J1a1a1-P58, as we see through our demographic analysis (Fig. 4), further complicates our possibility to find them in the early Holocene assemblages. Ne was increasing starting from the Chalcolithic and the Bronze Age periods (Fig. 4), the time when haplogroup J1a1a1-P58 appeared in the archaeological record of the Levant^22,46,61,62^. These are the oldest haplogroup J1a1a1-P58 members found so far in the world, further supporting its origin in the southern regions of West Asia. Thus, the apparent contradiction mentioned above, could be instead the expected outcome. In regions with an insufficient number of human fossils and low preservation of aDNA, demographic studies with contemporary samples are currently the only insight we have.

The expansion of haplogroup J1-M267 occurred over a long period—spanning the Chalcolithic, the Bronze Age, and the Iron Age. Many demographic events in the region could maintain the uninterrupted expansion of this haplogroup. During the Chalcolithic and the Bronze Age, people were moving intensively across West Eurasia^21,22,57,63,64^. At this time, the Levantine Neolithic ancestry increased in the northern areas of West Asia^22^, where the shared Caucasus hunter-gatherer/Iranian Neolithic ancestry or the Anatolian Neolithic ancestry were prevalent before. At the beginning of the population expansion, people belonging to haplogroup J1a1a1-P58, probably migrated also to the northern regions of West Asia and Europe from the Arabian Peninsula, southern Mesopotamia, and the Levant. Therefore, in the case of West Asia this evidence—based on the TMRCAs of the shared J1a1a1-P58 branches—mirrors that of genome-wide ancestry^22^. The migration of the J1a1a1-P58 lineages, though, was less pronounced towards the northern regions of West Asia and Europe, since the frequency of this haplogroup and the number of such branches are low there. During this time and especially thereafter, the spread within the Arabian Peninsula, southern Mesopotamia, and the southern Levant was more intense, resulting in a large number of local branches and the high frequency we find today. This expansion resembles the spread of Afro-Asiatic languages in West Asia^65^. Both the spread of J1a1a1-P58 and Afro-Asiatic languages could have been caused by the change of climatic conditions and the emergence of arid pastoralism as suggested earlier^23^.

Haplogroup J1-M267 occurs frequently in North and East Africa (Fig. 1, Supplementary Table S7**)**. Unfortunately, our North African collection includes only specimens from Egypt, where the demographic history of haplogroup J1-M267, in general, follows the pattern found in the Levant and the Arabian Peninsula. The only difference is that we haven’t found deeply diverging J1a1a1-P58 lineages in Egypt. In central and western regions of North Africa haplogroup J1-M267 may have a different history. Studies with STR haplotypes, some of them also with combined SNP markers, have reported different lineages of haplogroup J1-M267 in East Africa, more specifically in Ethiopia, Sudan, and Somalia^8,23,37^. Here, we have found at least three distinct lineages there. One of them likely belongs to a rare non-J1a1a1-P58 branch—J1a1a2-ZS4393—found among the Yemenis. The other two lineages belong to haplogroup J1a1a1-P58. One of them belongs to the widespread J1a1a1a1a1a1a1-L858 branch. The other is rare, found only in Omanis, Yemenis, Kuwaitis, and Ethiopians indicating a possible source. These lineages correspond to one or more migration episodes from West Asia to Ethiopia. Additional data may answer the question about the number of successful dispersals from West Asia to East Africa.

Haplogroup J1-M267 occurs with a low frequency in Europe (~ 1.7%) (Fig. 1, Supplementary Table S7). The Mediterranean and southeastern regions contain more members of haplogroup J1-M267 than the other regions. The distribution was originally associated with the spread of farming from the Near East^37^. So far, no aDNA study has reported this haplogroup in the European Neolithic, Chalcolithic, or Bronze Age assemblages (Supplementary Table S3). It appeared in Mediterranean Europe only in the historical period^48,66^. Unexpectedly, a member of haplogroup J1-M267 is found among eastern hunter-gatherers from Karelia, Northeast Europe living ~ 8.3 kya^67^. This branch is absent in other ancient European hunter-gatherers (Supplementary Table S3). Unfortunately, we fail to put this sample in the context of the current haplogroup J1-M267 variation because of the poor quality of the DNA sequence. A recent study with the extant variation concludes that Asia Minor is less likely to be the source of the Greek and Italian haplogroup J1-M267 chromosomes as they do not coalesce together before they coalesce to the lineages from Turkey^31^. They propose a neighboring area—the Caucasus—as the putative source. The current distribution in Europe is most likely the result of complex demographic processes, involving various sources considering the different lineages found there. In our whole Y chromosome tree, European haplogroup J1-M267 lineages coalesce to others after ~ 5 kya. Therefore, the ancestors of the current haplogroup J1-M267 members migrated to Europe after the Neolithic, and, given the aDNA results, most likely also after the Bronze Age, since the earliest representative was found in the Punic period in Sardinia at ~ 2.4 kya^48,68^ (Supplementary Fig. S2, Supplementary Table S3).

Haplogroup J1-M267 occurs sporadically in South Asia (~ 0.7%) (Fig. 1, Supplementary Table S7). The populations from Pakistan bear the largest portion of this haplogroup there. Different lineages hint at the complex history of haplogroup J1-M267 in South Asia. It is interesting to find the pre-Holocene TMRCA of the J1a2-PH77 branch, which involves a Punjabi coalescing with samples from the populations of the Armenian Highland and the South Caucasus (Fig. 2, Supplementary Fig. S1, Supplementary Table S4). Another deeply diverged branch, J1b-F4306, has representatives from both extant and ancient^45^ South Asian populations (Supplementary Fig. S2, Supplementary Table S3). These shared branches signal early contacts between South Asia and West Asia found elsewhere^22,51,69,70^. Other lineages coalesce ~ 5 kya in the branches J1a1b-Z18375 and J1a1a1a1a1a1a1-L858. They point to more recent contacts with populations from different West Asian regions.

The Cohen-specific lineage of haplogroup J1-M267 was first described as a Cohen-specific STR haplotype^38^, called “the Cohen modal haplotype”. It was rejected at first^10^ and then confirmed by an extended STR repertoire^71^. This later study reports that 46.1% of all Cohens fall within this lineage. Subsequently, a Cohen-specific branch was also found in the phylogenetic tree of haplogroup J1-M267^29^. Here, we confirm this Cohen-specific branch in haplogroup J1-M267 as J1a1a1a1a1a1a2-B877 (Fig. 2, Supplementary Fig. S1). All Jewish lineages of haplogroup J1-M267 fall into the J1a1a1-P58 branch (Supplementary Fig. S1), which suggests their origin ultimately in the Levant. It is surprising to find two Jewish or close to Jewish J1a1a1-P58 lineages in the ancient Roman samples (~ 1.5–2.0 kya)^48^. This tells us about the migration of the Jewish people, at least of the bearers of the J1a1a1-P58 chromosomes, who travelled from the Levant to Europe via Italy, consistent with an earlier research^29^.

Studies explain the current distribution of haplogroup J1-M267 to be a result of the Arab conquests connected to the diffusion of Islam^12,35,37^. If this scenario would have been true in West Asia, then the phylogeny of haplogroup J1-M267 should have contained multiple coalescences between representatives of different Arab populations within the time, when the diffusion of Islam occurred, that is, in the last ~ 1.3 ky^12,35,37^. In reality, such coalescences occur mostly within the period of ~ 2 to ~ 5 kya (Supplementary File S1). Moreover, we don’t find a substantial increase of Ne after ~ 1.3 kya. These observations contradict the connection between the spread of this haplogroup and the spread of Islam in West Asia and Egypt, consistent with previous study^10^. Considering our sampling limitation, we avoid excluding the connection between the spread of Islam and haplogroup J1-M267 in central and western North Africa. But we argue that in West Asia the distribution of haplogroup J1-M267 was already shaped before the spread of Islam. This conclusion aligns with aDNA studies, reporting J1a1a1-P58 at least before ~ 2.5 kya in a wide area encompassing Syria in the north and Egypt in the south^22,46,61,62,72^.

## Conclusions

Y chromosome haplogroup J1-M267 evolved in the northern parts of West Asia around the LGM. A limited number of founders migrated south—to the Arabian Peninsula, the southern Levant, and southern Mesopotamia, where the J1a1a1-P58 branch evolved in the early Holocene. Haplogroup J1-M267 expanded during the Chalcolithic, the Bronze Age, and the Iron Age, coinciding with the spread of Afro-Asiatic languages combined with the diffusion of arid pastoralism in the desert regions of West Asia. The spread of Islam did not substantially affect the distribution of haplogroup J1-M267 in West Asia.

## Materials and methods

### Samples

We collected blood specimens from 2341 healthy unrelated males representing 16 populations from West Asia, Egypt, and East Africa. Patrilineal ancestors of the samples for at least two generations belong to the populations reported here. Informed consent was obtained from all participants in the study. All experimental procedures were carried out in accordance with the approved guidelines by the Research Ethics Committee of the University of Tartu. All experimental protocols were approved by the Research Ethics Committee of the University of Tartu (252/M-17). DNA was extracted with the published “salting out” method^73^. We refined the sub-haplogroup status of J1-M267 members of additional 2460 published^26,74^ samples representing 26 populations from West Asia. All haplogroup J1-M267 samples were determined to be in the derived state at either M267^14^ or M497^75^ marker.

In this study, we define West Asia as including the Levant, the Arabian Peninsula, Mesopotamia, Iran, Anatolia, the Armenian Highland, and the Caucasus. In Supplementary Table S7, we listed the populations from the Caucasus and the other regions separately for ease of reading.

#### Spatial frequency and diversity analyses

Spatial frequency and diversity analyses were performed with Surfer program (version 8, Golden Software, Inc., Golden, CO, USA), following the Kriging procedure^76^. The input data is represented in Supplementary Table S7. The maps were drawn in RStudio software^77,78^. The details are provided in Supplementary Methods.

### Whole high-coverage Y chromosome resequencing

Sixteen new samples were resequenced with the Illumina HiSeq 2500 platform following Y chromosome capture using a proprietary capture protocol available at Gene by Gene (Family Tree DNA) using the commercially available “BigY” service (https://learn.familytreedna.com/wp-content/uploads/2014/08/BIG_Y_WhitePager.pdf). Its targeted enrichment design utilizes 67,000 capture probes for sequencing more than 10 Mbp in the non-recombining male-specific parts of the Y chromosome at > 60× coverage. The samples were selected to include as many different lineages as possible, especially those that according to initial phylogenies coalesce deeply in whole haplogroup J1-M267 and its J1a1a1-P58 branch. To reveal phylogenetic positions of the available samples we genotyped informative SNP markers. Among many similar candidates, we selected those that differ by STR haplotypes. The genotyping and STR haplotyping information are presented later in this section. Sample information is provided in Supplementary Table S1**.**

### Whole high-coverage Y chromosome data collection

We selected only the high-coverage Y chromosome genomes resequenced with next-generation sequencing technologies targeting over 9 Mb regions of the chromosome (Supplementary Table S1). We followed all required security guidelines. If fastq reads were unavailable in the public servers we converted BAM or CRAM genome files into fastq read files using SAMtools^79^ v1.9 and bedtools v2.24^80^.

#### Reads mapping and multi-sample Y chromosome variants calling

These procedures of the genomes resequenced by Illumina platform were carried out as have been described earlier^29^, following the best practices recommended by the SAMtools^79^ developers (http://www.htslib.org/workflow). The reads were mapped to the GRCh37 reference assembly. Called variants then were combined with Y chromosome variants extracted from published high-coverage whole genomes resequenced by Complete Genomics technology (Mountain View, California) (Supplementary Table S1). The procedure for mapping reads and calling multi-sample Y chromosome variants is described in detail in Supplementary Methods.

### Variants filtering

The region mask we used is based on the published callable regions^81^. These regions were supplemented with published^15^ high-quality regions. This filter concentrates on parts of Y chromosome reachable with NGS (the ‘re-mapping filter’) and minimizes the platform bias after datasets are merged. After construction of initial phylogenetic tree, we noticed some inconsistencies, which we corrected by excluding regions based on the following considerations. First, we saw that the terminal branches are rich in two or more subsequent (within 50 base pairs) singletons. We have excluded all that regions. Second, we have excluded all the regions containing discrepant SNPs between the genomes of the same individuals resequenced more than once or between the genomes of paternally related individuals. Out of several genomes of the same or paternally related individual we left for the downstream analyses the one with the least number of no-calls. Third, we have excluded the regions with recurrent SNPs occurring in three and more branches in phylogeny composed with samples sequenced with the same platform. Fourth, we have excluded the regions with missing data in more than 10% of samples. Moreover, we have excluded also the regions between two > 10% N sites if they are placed nearby and there was no any high-quality variant there. In the end, we recover 9,429,728 bases of the male-specific region of the Y chromosome (Supplementary Table S6). More than one alternative alleles in the same position are considered as independent events. We have not performed imputation as the platform-specific differences are expected to be negligible due to the region mask we used. This conclusion is supported by the low variation of mutation rates among the branches in the Bayesian phylogenetic analysis (0.0932, 95% HPD = 0.013–0.161).

### Phylogenetic tree reconstructions

We reconstructed whole high-coverage Y chromosome haplogroup J1-M267 phylogeny with two different methods—maximum-likelihood (ML) and Bayesian Markov Chain Monte Carlo (MCMC) approach. The details of ML tree reconstruction are provided in Supplementary Methods. The tree can be found in Supplementary Fig. S1. The polymorphic positions and their annotations are represented in Supplementary Table S2.

#### Coalescence time estimates

These were determined simultaneously with the tree reconstruction using Bayesian MCMC approach implemented in BEAST v1.10.4 software^82^. For proper rooting, we have added one sample of haplogroup J2-M172. Two parallel analyses were run with different random number seeds. Thirty million chains were run for every analysis. Haplogroup J1-M267 coalescence time estimates were computed with normally distributed age prior of 18,741 ± 1874 years for the node in our phylogeny resembling the whole haplogroup J1-M267 MRCA published previously^15^. We chose the calibration method with a node age, to avoid introducing bias due to reported substantial Y chromosome mutation rate variation among different haplogroups^39,40,83,84^. We used no other topology constraint in our analyses. The details about the analysis and priors are in Supplementary Methods.

The results were manually checked with Tracer v1.7 software^85^. Good convergence was achieved according to effective sample size (ESS) values well above 200 for all parameters. The results of the two parallel chains were combined using the LogCombiner software^86^ leaving the first 10% chains of every run as burn-in. The maximum clade credibility (MCC) tree was generated with TreeAnnotator^86^ leaving out the first 10% trees of every run as burn-in. We summarized the node heights with the posterior median values. The MCC tree was visualized in RStudio software^77,78^. The details are provided in Supplementary Methods.

#### Bayesian phylogeography

Bayesian phylogeographic analysis in continuous space was performed according to the published method^49,50^. It was used to reveal the ancestral location and spatial dynamics of the viruses in continuous space^50,87^ as well as in linguistic studies^88^. We have chosen 122 and 103 whole Y chromosome sequences in the haplogroups J1-M267 and J1a1a1-P58 analyses, respectively, which recover the widest possible distribution of the haplogroups. We included the genomes for which the geographic coordinates could be provided. We left out the genomes from the Americas. For computational purposes, we remove one out of two samples from the same population coalescing within the recent ~ 1000 years. This approach may alter the Ne in the recent 1000 years, but for this particular analysis, Ne in the recent 1000 years is not essential. The analyses were done with BEAST v1.10.4 software^82^. The molecular clock, site, and tree models and priors were similar to those used in the Bayesian phylogenetic analysis described before. The coalescence time estimation in the J1a1a1-P58 analysis was done by providing normally distributed prior with 9524 ± 952 years to the root node. This age was estimated for the J1a1a1-P58 branch in our study. The Brownian random walk (BRW) model^49,89^ was used as the diffusion model in continuous space. We have run three sets of 300,000,000 chains for every haplogroup ensuring ESS values well above 200. The MCC tree was generated with -hpd2D 0.8 flag to summarize 80% HPD area for the tree nodes. The uncertainties of the MCC tree node locations were visualized with spreaD3_v0.9.7.1rc.jar software^90^ using the world map in the “geojson” format as the base map downloaded from https://github.com/Stefie/geojson-world.

#### The demographic history reconstruction of haplogroup J1-M267

This was performed with Bayesian skyline analysis framework^91^. The analysis set-up was similar to the Bayesian phylogenetic reconstruction analysis described before, except that here we took only haplogroup J1-M267 sequences. The dynamics of Ne through time was estimated with Tracer v1.7 software^85^. Runs with different group sizes ranging between 3 and 10 resulted in similar phylodynamic curves. The Bayesian skyline plot was drawn with the analysis of 5 groups (default value in BEAST v1.10.4 software^82^) using the R software^77^ with the basic packages. We have used 31 years as the average per generation time for human males^92^.

### Annotation

Haplogroup J1-M267 ML tree serves the phylogenetic basis for our annotations. We annotated the branches according to the published principles^14^. The names were supplemented with the defining marker names. We preferentially use the names of the markers genotyped in our study. We almost always extend the names of the major branches by “a” or “1” suffixes, while other letters and numbers we use for the minor branches. Branch annotations are represented in Supplementary Table S2.

### Genotyping of the phylogenetically informative SNP markers

The genotyping was performed to assign every haplogroup J1-M267 sample available to us to the known branches of the reconstructed tree. In our study, we tend to genotype SNP markers residing on the deeper branches in haplogroups J1-M267 and J1a1a1-P58 to ensure that all of the deeply divergent variants are revealed, and most of them we sent for the high-coverage whole Y chromosome sequencing. SNP marker candidates among the phylogenetically equivalent options were chosen based on the primer parameters. We also ensured the SNPs are non-recurring. Details of the primer design process are provided in Supplementary Methods. The allele statuses were identified by direct Sanger sequencing or restriction fragment length polymorphism (RFLP) analysis. Overall, 39 haplogroup J1-M267 SNP markers were genotyped in 947 members of this haplogroup from 43 populations. For 58 samples we obtained both genotyping and high-coverage whole Y chromosome sequence data, and the genotypes called from high-coverage whole Y chromosome sequences agree with the genotypes revealed by Sanger sequencing or RFLP. Primer specifications and the genotypes are provided in Supplementary Tables S4 and S5, respectively.

### Y chromosome STR genotyping and diversity calculations

We genotyped 17 or 23 Y-STRs in 540 haplogroup J1-M267 samples using the AmpFlSTR Yfiler kit (Applied Biosystems) or the PowerPlex 23 kit (Promega Corporation), respectively, according to manufacturer recommendations. The details of STR genotyping are provided in Supplementary Methods. The STR haplotypes of the new samples are shown in Supplementary Table S8.

We calculated STR diversity estimates with the following 8 loci—DYS19, DYS389I, DYS389II, DYS390, DYS391, DYS392, DYS393, and DYS439 to incorporate also the published data. The details are provided in Supplementary Methods.

#### The ancient haplogroup J1-M267 representatives’ affiliation

The ancient haplogroup J1-M267 representatives’ affiliation to the reconstructed phylogeny was performed by revealing the alleles of 4292 SNPs in the BAM genome files. We downloaded published genome BAM files of the samples reported to belong either to haplogroup J1-M267 or to uncertain haplogroup J branch in the original publication (Supplementary Table S3). Initially, we called all Y chromosome variants with SAMtools^79^, applying the following command “samtools mpileup -E -vu -d 500 -s -f hs37d5.fa.bgz -r Y -O -skip-indels -output file.vcf file.bam”. Afterwards, all 4292 variable positions were searched in the generated VCF file. Every SNP with the derived state was manually looked in the genome and variant files for the quality measures. We report the sample to be a representative of the branch if the branch and its upstream branches are supported with derived alleles. We also ensured that the SNPs defining the sister branches to have ancestral alleles. The results are represented in Supplementary Fig. S2 and Supplementary Table S3.

## Supplementary Information

Supplementary Figure S1.

Supplementary Figure S2.

Supplementary File S1.

Supplementary Methods.

Supplementary Note.

Supplementary Tables.

Supplementary File S1 Legend.

## Data Availability

The data generated in the current study are available in the European Nucleotide Archive (ENA) at EMBL-EBI, (https://www.ebi.ac.uk/ena/browser/view/PRJEB41598) under accession PRJEB41598.
