# Supplementary material for: Origin and diffusion of human Y chromosome haplogroup J1-M267
Source: Sci Rep. 2021 Mar 23;11:6659. doi: 10.1038/s41598-021-85883-2 (PMC7987999; doi:10.1038/s41598-021-85883-2)
Supplement: Supplementary file 4 — Supplementary Methods. [file 41598_2021_85883_MOESM4_ESM.docx]

**Supplementary Methods**

**Origin and diffusion of human Y chromosome haplogroup J1-M267**

Hovhannes Sahakyan^1,2,†,*^, Ashot Margaryan^2,3,†^, Lauri Saag^1^, Monika Karmin^1,4^, Rodrigo Flores^1^, Marc Haber^5^, Alena Kushniarevich^1^, Zaruhi Khachatryan^2^, Ardeshir Bahmanimehr^2,11^, Jüri Parik^1,6^, Tatiana Karafet^7^, Bayazit Yunusbayev^1,8^, Tuuli Reisberg^9^, Anu Solnik^1^, Ene Metspalu^1^, Anahit Hovhannisyan^2^, Elza K Khusnutdinova^8,10^, Doron M Behar^1^, Mait Metspalu^1^, Levon Yepiskoposyan^2^, Siiri Rootsi^1,†^, and Richard Villems^1,6,†^

^1^Estonian Biocentre, Institute of Genomics, University of Tartu, Tartu, 51010, Estonia.

^2^Laboratory of Evolutionary Genomics, Institute of Molecular Biology of National Academy of Sciences of the Republic of Armenia, Yerevan, 0014, Armenia.

^3^Lundbeck Foundation, GeoGenetics Centre, Department of Biology, University of Copenhagen, Copenhagen K, 1350, Denmark.

^4^Statistics and Bioinformatics Group, Institute of Fundamental Sciences, Massey University, Palmerston North, Manawatu, 4442, New Zealand.

^5^Institute of Cancer and Genomic Sciences, University of Birmingham, Birmingham, B15 2TT, UK.

^6^Department of Evolutionary Biology, Institute of Cell and Molecular Biology, University of Tartu, Tartu 51010, Estonia.

^7^ARL Division of Biotechnology, University of Arizona, Tucson, Arizona, 85721, USA.

^8^Department of Genetics and Fundamental Medicine of Bashkir State University, Ufa, 450076, Bashkortostan, Russia.

^9^Core Facility, Institute of Genomics, University of Tartu, Tartu, 51010, Estonia

^10^Institute of Biochemistry and Genetics of Ufa Federal Research Center of the Russian Academy of Sciences, Ufa, 450054, Russia.

^11^Present address: Thalassemia and Haemophilia Genetic PND Research Center, Dastgheib Hospital, Shiraz University of Medical Sciences, Shiraz, 71456–83769, Iran.

^†^These authors contributed equally to this work.

^*^Corresponding author: E-mail: [hovhannes.sahakyan@ut.ee](mailto:hovhannes.sahakyan@ut.ee), [hovhannesuk@yahoo.co.uk](mailto:hovhannesuk@yahoo.co.uk)

**Reads mapping** **and multi-sample Y chromosome variants calling** of the genomes resequenced by Illumina platform were carried out as have been described earlier^1^, following the best practices recommended by the SAMtools^2^ developers (http://www.htslib.org/workflow). Reads were mapped with BWA-MEM^3,4^ to the GRCh37 human reference assembly, ‘decoy’ version obtained from the 1000 Genomes Project^5^. Duplicate reads have been removed with picard-tools-2.0.1 (http://broadinstitute.github.io/picard). Indel realignment was done with GATK-3.5^6^. Finally, multi-sample base calling was performed by SAMtools^2^ and BCFtools^7^. All parameters were published earlier^1^. All Y chromosome genomes sequenced by Illumina platform were mapped and called using the same script starting from the raw fastq reads. These variants then were combined with Y chromosome variants extracted from published high coverage whole genomes resequenced by Complete Genomics technology (Mountain View, California) (**Supplementary Table S1. Whole Y chromosomes**).

**Spatial frequency and diversity** maps were drawn in RStudio software^8,9^ using the following packages –“lattice”^10^, “sp”^11,12^, “raster”^13^, “rgdal”^14^, “rgeos”^15^, and “classInt”^16^ in addition to the basic packages.

**Maximum likelihood (ML) tree reconstructions**. ML tree was reconstructed with RAxML software version 7.3.2^17^, using generalized time reversible (GTR) substitution matrix^18^, rapid bootstrapping (n=200) and subsequent ML search. For proper rooting we included two members of haplogroups J2-M172 and I each. All identified variants were annotated based on this phylogeny using in-house scripts followed by manual curation. The haplogroup J1-M267 ML tree can be found in **Supplementary Figure S1.** The polymorphic positions and their annotations are represented in **Supplementary Table S2**.

**Priors for Bayesian MCMC analysis for phylogeny reconstruction and coalescence time estimation**. For the site model we used GTR substitution model^18^ and Gamma site heterogeneity model^19^ with 4 categories. We used the uncorrelated relaxed log-normal clock model^20^ in the analysis. Log-normal prior distribution for the ucld.mean was given with M=5.0e^-8^ and S=5.0e^-8^ in real space. And for ucld.stdev we gave also log-normally distributed prior with M=5.0e^-9^ and S=5.0e^-9^ in real space. Bayesian skyline model^21^ was used as the tree model with group sizes of 5. We have chosen the piecewise-linear approach for smoothing the population dynamics. Uniform distribution bounded by 0 and 1.0e15 was given as the skyline.popSize prior. In all analyses with BEAST v1.10.4 we used BEAGLE library v3.1.2^22^ for accelerated, parallel likelihood evaluation.

**The MCC tree** was visualized in RStudio software^8,9^ using “ggtree”^23,24^, “ape”^25^, “treeio”^26^, “reshape2”^27^, “ggplot2”^28^, “ggstance”^29^ packages in addition to the basic packages.

**Primer design** was carried out with Primer3plus software^30–32^ with suggested^33,34^ settings. The primer pairs’ qualities were initially assessed with Primer-BLAST online software^35^. The primer pairs with the least number of putative products were selected. The real performances were assessed with the electrophoresis of the Polymerase chain reaction products and by direct Sanger sequencing of them.

**STR genotyping and diversity estimation**. We separated the amplified fragments with an ABI PRISM 3130xl Genetic Analyzer (Applied Biosystems), and analyzed the results with the ABI PRISM program GeneMapper 4.0 (Applied Biosystems). The following Y-STR loci were genotyped with the Y-Filer Kit – DYS19, DYS389I, DYS389II, DYS390, DYS391, DYS392, DYS393, DYS439, DYS385a, DYS385b, DYS437, DYS438, DYS448, DYS456, DYS458, DYS635, and Y GATA H4. The following six Y-STRs are genotyped with the PowerPlex 23 kit in addition to the above mentioned 17 Y-STRs – DYS481, DYS533, DYS549, DYS570, DYS576, DYS643. We have genotyped also DYS388 and DYSA7.2 (also called DYS461) Y-STRs separately^36,37^. These two STRs are informative in the context of haplogroup J^38,39^.

We calculated STR diversity estimates with the following 8 loci – DYS19, DYS389I, DYS389II, DYS390, DYS391, DYS392, DYS393, and DYS439 to incorporate also the published data. Using Arlequin v3.5^40^ we estimated the average gene diversity over the eight STR loci computed as the probability that two randomly chosen homologous sites are different^41,42^. We removed all individuals with more than one missing STR loci and only considered populations with the sample size of above 10 individuals, resulting in a total of n=2121 individuals across 41 populations for the whole J1-M267 analysis. We calculated STR diversities also separately for J1-M267(xJ1a1a1-P58) and J1a1a1-P58. For the J1-M267(xJ1a1a1-P58) analysis we used n=251 individuals across 14 populations, and for the J1a1a1-P58 analysis we used n=1418 individuals across 28 populations.

**Bibliography**

1. Behar, D. M. *et al.* The genetic variation in the R1a clade among the Ashkenazi Levites’ Y chromosome. *Sci. Rep.* **7**, 14969 (2017).

2. Li, H. *et al.* The Sequence Alignment/Map format and SAMtools. *Bioinformatics* **25**, 2078–2079 (2009).

3. Li, H. Aligning sequence reads, clone sequences and assembly contigs with BWA-MEM. *ArXiv13033997v2 Q-BioGN* (2013).

4. Li, H. & Durbin, R. Fast and accurate long-read alignment with Burrows–Wheeler transform. *Bioinformatics* **26**, 589–595 (2010).

5. The 1000 Genomes Project Consortium. A global reference for human genetic variation. *Nature* **526**, 68–74 (2015).

6. McKenna, A. *et al.* The Genome Analysis Toolkit: A MapReduce framework for analyzing next-generation DNA sequencing data. *Genome Res.* **20**, 1297–1303 (2010).

7. Danecek, P. *et al.* The variant call format and VCFtools. *Bioinformatics* **27**, 2156–2158 (2011).

8. R CoreTeam. *R: A language and environment for statistical computing.* (R Foundation for Statistical Computing, 2019).

9. RStudio Team. *RStudio: Integrated Development Environment for R*. (RStudio, Inc., 2019).

10. Sarkar, D. *Lattice: Multivariate Data Visualization with R*. (Springer-Verlag, 2008). doi:10.1007/978-0-387-75969-2.

11. Bivand, R. S., Pebesma, E. J. & Virgilio, G.-R. *Applied spatial data analysis with R, Second edition.* (Springer, 2013).

12. Pebesma, E. J. & Bivand, R. S. Classes and methods for spatial data in R. *R News* **5**, 9–13 (2005).

13. Hijmans, R. J. *raster: Geographic Data Analysis and Modeling.* (2019).

14. Bivand, R. S., Keitt, T. & Rowlingson, B. *rgdal: Bindings for the ‘Geospatial’ Data Abstraction Library.* (2019).

15. Bivand, R. S. & Rundel, C. *rgeos: Interface to Geometry Engine - Open Source ('GEOS’).* (2019).

16. Bivand, R. S. *classInt: Choose Univariate Class Intervals.* (2019).

17. Stamatakis, A. RAxML version 8: a tool for phylogenetic analysis and post-analysis of large phylogenies. *Bioinformatics* **30**, 1312–1313 (2014).

18. Tavaré, S. Some probabilistic and statistical problems in the analysis of DNA sequences. in *American Mathematical Society: Lectures on Mathematics in the Life Sciences* vol. 17 57–86 (Amer Mathematical Society, 1986).

19. Yang, Z. Maximum likelihood phylogenetic estimation from DNA sequences with variable rates over sites: Approximate methods. *J. Mol. Evol.* **39**, 306–314 (1994).

20. Drummond, A. J., Ho, S. Y. W., Phillips, M. J. & Rambaut, A. Relaxed phylogenetics and dating with confidence. *PLoS Biol* **4**, e88 (2006).

21. Drummond, A. J., Rambaut, A., Shapiro, B. & Pybus, O. G. Bayesian coalescent inference of past population dynamics from molecular sequences. *Mol. Biol. Evol.* **22**, 1185–1192 (2005).

22. Ayres, D. L. *et al.* BEAGLE: An Application Programming Interface and High-Performance Computing Library for Statistical Phylogenetics. *Syst. Biol.* **61**, 170–173 (2012).

23. Yu, G., Lam, T. T.-Y., Zhu, H. & Guan, Y. Two Methods for mapping and visualizing associated data on phylogeny using ggtree. *Mol. Biol. Evol.* **35**, 3041–3043 (2018).

24. Yu, G., Smith, D. K., Zhu, H., Guan, Y. & Lam, T. T.-Y. ggtree: an r package for visualization and annotation of phylogenetic trees with their covariates and other associated data. *Methods Ecol. Evol.* **8**, 28–36 (2017).

25. Paradis, E. & Schliep, K. ape 5.0: an environment for modern phylogenetics and evolutionary analyses in R. *Bioinformatics* **35**, 526–528 (2019).

26. Wang, L.-G. *et al.* Treeio: an R package for phylogenetic tree input and output with richly annotated and associated data. *Mol. Biol. Evol.* **37**, 599–603 (2020).

27. Wickham, H. Reshaping data with the reshape package. *J. Stat. Softw.* **21**, 1–20 (2007).

28. Wickham, H. *Ggplot2: elegant graphics for data analysis*. (Springer, 2009).

29. Henry, L., Wickham, H. & Chang, W. *ggstance: horizontal ‘ggplot2’ components*. (2019).

30. Rozen, S. & Skaletsky, H. Primer3 on the WWW for General Users and for Biologist Programmers. in *Bioinformatics Methods and Protocols* vol. 132 365–386 (Humana Press, 1999).

31. Untergasser, A. *et al.* Primer3—new capabilities and interfaces. *Nucleic Acids Res.* **40**, e115–e115 (2012).

32. Untergasser, A. *et al.* Primer3Plus, an enhanced web interface to Primer3. *Nucleic Acids Res.* **35**, W71–W74 (2007).

33. Koressaar, T. & Remm, M. Enhancements and modifications of primer design program Primer3. *Bioinformatics* **23**, 1289–1291 (2007).

34. SantaLucia, J. A unified view of polymer, dumbbell, and oligonucleotide DNA nearest-neighbor thermodynamics. *Proc. Natl. Acad. Sci.* **95**, 1460–1465 (1998).

35. Ye, J. *et al.* Primer-BLAST: A tool to design target-specific primers for polymerase chain reaction. *BMC Bioinformatics* **13**, 134 (2012).

36. Kayser, M. *et al.* Applications of microsatellite-based Y chromosome haplotyping. *Electrophoresis* **18**, 1602–1607 (1997).

37. White, P. S., Tatum, O. L., Deaven, L. L. & Longmire, J. L. New, Male-Specific Microsatellite Markers from the Human Y Chromosome. *Genomics* **57**, 433–437 (1999).

38. Cinnioğlu, C. *et al.* Excavating Y-chromosome haplotype strata in Anatolia. *Hum. Genet.* **114**, 127–148 (2004).

39. Yunusbayev, B. *et al.* The Caucasus as an asymmetric semipermeable barrier to ancient human migrations. *Mol. Biol. Evol.* **29**, 359–365 (2012).

40. Excoffier, L. & Lischer, H. E. L. Arlequin suite ver 3.5: a new series of programs to perform population genetics analyses under Linux and Windows. *Mol. Ecol. Resour.* **10**, 564–567 (2010).

41. Nei, M. *Molecular evolutionary genetics*. (Columbia University Press, 1987).

42. Tajima, F. Evolutionary relationship of DNA sequences in finite populations. *Genetics* **105**, 437–460 (1983).
