# Supplementary material for: Origin and diffusion of human Y chromosome haplogroup J1-M267
Source: Sci Rep. 2021 Mar 23;11:6659. doi: 10.1038/s41598-021-85883-2 (PMC7987999; doi:10.1038/s41598-021-85883-2)
Supplement: Supplementary file 5 — Supplementary Note. [file 41598_2021_85883_MOESM5_ESM.docx]

**Supplementary Note**

**Origin and diffusion of human Y chromosome haplogroup J1-M267**

Hovhannes Sahakyan^1,2,†,*^, Ashot Margaryan^2,3,†^, Lauri Saag^1^, Monika Karmin^1,4^, Rodrigo Flores^1^, Marc Haber^5^, Alena Kushniarevich^1^, Zaruhi Khachatryan^2^, Ardeshir Bahmanimehr^2,11^, Jüri Parik^1,6^, Tatiana Karafet^7^, Bayazit Yunusbayev^1,8^, Tuuli Reisberg^9^, Anu Solnik^1^, Ene Metspalu^1^, Anahit Hovhannisyan^2^, Elza K Khusnutdinova^8,10^, Doron M Behar^1^, Mait Metspalu^1^, Levon Yepiskoposyan^2^, Siiri Rootsi^1,†^, and Richard Villems^1,6,†^

^1^Estonian Biocentre, Institute of Genomics, University of Tartu, Tartu, 51010, Estonia.

^2^Laboratory of Evolutionary Genomics, Institute of Molecular Biology of National Academy of Sciences of the Republic of Armenia, Yerevan, 0014, Armenia.

^3^Lundbeck Foundation, GeoGenetics Centre, Department of Biology, University of Copenhagen, Copenhagen K, 1350, Denmark.

^4^Statistics and Bioinformatics Group, Institute of Fundamental Sciences, Massey University, Palmerston North, Manawatu, 4442, New Zealand.

^5^Institute of Cancer and Genomic Sciences, University of Birmingham, Birmingham, B15 2TT, UK.

^6^Department of Evolutionary Biology, Institute of Cell and Molecular Biology, University of Tartu, Tartu 51010, Estonia.

^7^ARL Division of Biotechnology, University of Arizona, Tucson, Arizona, 85721, USA.

^8^Department of Genetics and Fundamental Medicine of Bashkir State University, Ufa, 450076, Bashkortostan, Russia.

^9^Core Facility, Institute of Genomics, University of Tartu, Tartu, 51010, Estonia

^10^Institute of Biochemistry and Genetics of Ufa Federal Research Center of the Russian Academy of Sciences, Ufa, 450054, Russia.

^11^Present address: Thalassemia and Haemophilia Genetic PND Research Center, Dastgheib Hospital, Shiraz University of Medical Sciences, Shiraz, 71456–83769, Iran.

^†^These authors contributed equally to this work.

^*^Corresponding author: E-mail: [hovhannes.sahakyan@ut.ee](mailto:hovhannes.sahakyan@ut.ee), [hovhannesuk@yahoo.co.uk](mailto:hovhannesuk@yahoo.co.uk)

**Detailed phylogeny of haplogroup J1-M267**

Haplogroup J1-M267 is defined by 103 SNP markers in our reconstructed phylogeny (Supplementary Figure S1, Supplementary Table S2). The deepest split in the tree yields in two branches – J1a-PF4644 and J1b-F4306 (Figure 2). The TMRCA of J1b-F4306 is ~13.2 kya (95% HPD = 10.2-16.6 kya) (Table 1). Interestingly, the ~13 thousand-year-old hunter-gatherer^1^ from the South Caucasus belongs to this branch. Three ancient humans found in Swat Valley in Pakistan (~0.6-2 kya)^2^ also belong to J1b-F4306 (Supplementary Figure S2, Supplementary Table S3). Our comprehensive genotyping analysis shows that J1b-F4306 branch is rare among contemporary populations (Figure 2). It occurs in Armenians, Balochis from Pakistan, and Colombians. Three specimens one from every population of Egyptians, Abkhazians and Kabardinians have the ancestral allele of PF4644 marker, therefore, we consider them as belonging to J1-F4306 branch (Supplementary Table S4). The absence of this branch in the southern regions of West Asia and presence of only a single occurrence of J1-F4306 in Egypt can be explained by the recent resettlement of the Caucasus people to the southern territories of the Ottoman Empire^3^. In Colombians J1b-F4306 chromosomes and the other haplogroup J1-M267 lineages could have been derived from different West Asian populations in parallel with genome-wide ancestry^4^.

J1a-PF4644 branch splits into J1a1-Z2217 and J1a2-PH77. J1a2-PH77 further splits into two branches J1a2a-PH351 and J1a2b-B2039 (Figure 2). J1a2a-PH351 branch is found only in the Caucasus, the Armenian Highland, and northwestern Iran (Supplementary Figure S1, Supplementary Table S4). J1a2b-B2039 branch is represented with a single Punjabi individual from Pakistan, and possibly, also with one individual from northwestern Iran. The TMRCA of J1a2-PH77 branch resembles to that of J1b-F4306 and equals to ~12.8 kya (95% HPD = 9.9-15.6 kya) (Table 1). Hence, both the time of divergence and the geographic distribution of J1a2-PH77 mirror that of J1b-F4306.

J1a1-Z2217 further splits into J1a1a-Z2359 and J1a1b-Z18375 (Figure 2). J1a1b-Z18375 branch coalesces ~6.5 kya (95% HPD = 5.0-8.0 kya) (Table 1) and is found with high frequencies again in the Caucasus and the neighboring regions (Supplementary Figure S1, Supplementary Table S4). Small number of representatives are found in Assyrians, Iraqis, Lebanese, Jordanians, Bedouins, Yemenis, Egyptians, and Sindhis. This branch constitutes the aforementioned haplogroup J1-M267 frequency peak in the Northeast Caucasus. All the members of this branch genotyped for DYS388 STR, have alleles with 14 or less repeats as noticed also previously^5,6^. In aDNA studies J1a1b-Z18375 branch is observed in the Chalcolithic Anatolia (5.3 kya)^7^, the Bronze Age Caucasus (~4.9 kya)^8^ and Anatolia (4.4 kya)^9^, and the Iron Age Levant (~2.5-2.8 kya)^7^ (Supplementary Table S3).

J1a1a-Z2359 branch divides into two branches – the well-known major J1a1a1-P58 and J1a1a2-ZS4393. J1a1a-Z2359 is found in the Chalcolithic northern Levant close to Anatolia and among Copper Age/Eneolithic and Early Bronze Age individuals in Central Asia adjacent to present-day Iranian border^2,10^. The northern Levantine/Anatolian individuals share more than half of their autosomal DNA ancestry with either Anatolian or Iranian Neolithic populations. The individuals from Central Asia share over 78% of their autosomal ancestry with Iranian Mesolithic and early farmer groups. J1a1a-Z2359 is found in the Iron Age Kazakhstan (~2.6 kya)^11^. The early presence of J1a1a-Z2359 branch to the north of the northern West Asian regions suggests the origin of this branch somewhere nearby.

Our phylogeographic survey has identified samples bearing the derived allele for Z2217 marker defining J1a1-Z2217 branch and ancestral alleles for the genotyped markers defining J1a1b-Z18375 and J1a1a1-P58 branches. These samples occur in a wide geographic area encompassing West Asia and East Africa (Supplementary Table S4). In East Africa 40% of haplogroup J1-M267 lineages belongs to this group. These samples are likely linked to J1a1a2-ZS4393 branch, composed by two recently diverged samples from Yemen^12^. Alternatively, these samples could represent instead a paragroup.

J1a1a1-P58 represents the major branch of haplogroup J1-M267 (Figure 1, Figure2, Supplementary Figure S1, Supplementary File S1). The frequency peaks in the Arabian Peninsula, southern Mesopotamia, and the southern Levant. Moderate frequencies occur in the northern parts of West Asia, North Africa, and East Africa. Lower frequencies occur in Europe, Central Asia, and South Asia. It is defined by 23 SNPs and coalesces 9.5 kya (95% HPD = 7.4-11.7 kya) (Table 1, Supplementary File S1). It splits off into J1a1a1a-Z1885 and J1a1a1b-Z18315 branches. One sample from northwestern Iran have the derived allele for P58 marker and the ancestral allele for all genotyped markers defining both downstream branches – J1a1a1a-Z1885 and J1a1a1b-Z18315. This could be a single representative of a putative rare lineage, which splits earlier than all the other sampled J1a1a1-P58 lineages. Alternatively, it could be a member of either J1a1a1a-Z1885 or J1a1a1b-Z18315 branch, but had diverged earlier than the genotyped mutations occurred. In all cases we would expect such a sample to be from the southern regions of West Asia as the other samples from the deep splitting lineages in J1a1a1-P58. Considering the single occurrence of such a sample, this can be a recent migrant from the south to the north of West Asia.

J1a1a1b-Z18315 is a minor branch. It is virtually absent in the northern parts of West Asia (Supplementary Table S4). It consists of two sub-branches: J1a1a1b1-L817 – found in Ashkenazi Jews and European populations, and J1a1a1b2-ZS5392 – found in populations from the Arabian Peninsula, southern Mesopotamia, and East Africa. The TMRCAs of J1a1a1b-Z18315, J1a1a1b1-L817, and J1a1a1b2-ZS5392 equal to 7.6 kya (95% HPD = 5.7-9.4 kya), 1.5 kya (95% HPD = 0.9-2.1 kya), and 3.8 kya (95% HPD = 2.7-4.9 kya), respectively. J1a1a1b1-L817 branch is most likely of the West Asian origin as J1a1a1b2-ZS5392 branch. The TMRCA of J1a1a1b1-L817 fits to the other Ashkenazi Jewish Y chromosome founders’ age^13^. A 300-500 CE individual from Rome belongs to this branch^14^, sharing derived alleles of 13 out of 16 defining SNPs sequenced in the ancient sample. Compared with the other men from this period, this ancient individual is closer to the Levantine and Jewish populations.

J1a1a1a-Z1885 branch coalesces ~9.0 kya (95% HPD = 7.0-11.1 kya) (Supplementary File S1). It remains with an unresolved hard polytomy in the ML tree splitting into three branches (Supplementary Figure S1). J1a1a1a3-B2146 is represented with a single sample from Jordan. We found one more sample from Yemen in the genotyping dataset putatively belonging to this branch. Another member is found in the published low-coverage genome dataset from Lebanon^15^. J1a1a1a2-ZS1280 is a rare lineage in our datasets found only in Yemen. It coalesces ~4.2 kya (95% HPD = 2.9-5.6 kya). A Bronze Age member of J1a1a1a2-ZS1280 branch is found in the southern Levant (3.6 kya)^16^.

J1a1a1a1-L2320 branch coalesces ~8.3 kya (95% HPD = 6.5-10.3 kya) (Supplementary File S1). It splits into J1a1a1a1a-Z1853 and J1a1a1a1b-B2062 branches (Figure2, Supplementary Figure S1). The latter is represented in our datasets with only a single representative from Saudi Arabia. Two Middle Bronze Age representatives (3.4-3.6 kya)^16^ from the southern Levant belong to J1a1a1a1-L2320 branch.

J1a1a1a1a-Z1853 coalesces ~7.3 kya (95% HPD = 5.7-9.0 kya) (Table 1, Supplementary File S1). This branch preserves an important phylogeographic mark as the branch to which most of the J1a1a1-P58 lineages of the northern regions of West Asia belong to (branch with number 11 in Figure2, Supplementary Figure S1). Two Middle Bronze Age genomes from the Levant belong to this branch (Supplementary Table S3). The northern Levantine one (4.5 kya) is from the Ebla population that traces more autosomal DNA ancestry from the southern Levantine populations compared with its contemporaneous neighbors^10^. This individual is so far the oldest ancient member of haplogroup J1a1a1-P58. The other J1a1a1a1a-Z1853 individual (3.7 kya)^16^ is from southern Levant.

Unresolved hard polytomy in J1a1a1a1a-Z1853 consists of three branches (Supplementary Figure S1). A single individual from Armenia represents J1a1a1a1a3-B2147. J1a1a1a1a2-ZS3683 occurs in Armenians, Adyghes, and Kuwaitis (Supplementary Table S4). J1a1a1a1a1-L862 branch coalesces 6.2 kya (95% HPDs = 4.9-7.6 kya) (Supplementary File S1). Ancient members of this branch are found in the Middle Bronze Age to Iron Age southern Levant (3.8-3.3 kya)^15–17^, pre-Ptolemaic period Egypt (~2.7 kya)^18^ and Punic period Sardinia (~2.4 kya)^19^ (Supplementary Table S3).

Two branches of J1a1a1a1a1-L862 – J1a1a1a1a1a-Z2324 and J1a1a1a1a1b-Z18297 – coalesce ~6.1 kya (95% HPDs = 4.8-7.4 kya) and ~5.8 kya (95% HPDs = 4.5-7.2 kya), respectively (Supplementary File S1). The latter has geographically wide distribution among contemporary populations (Supplementary Table S4). It occurs in the northern and the southern parts of West Asia, in Egypt, and also in Europe, in Belarusians and in one individual from the USA with the paternal ancestry from Germany. One Sephardic Jewish Cohen also belongs to this branch. J1a1a1a1a1a-Z2324 is found in three Bronze Age individuals (~3.4-4.1 kya)^16,20^ from the southern Levant (Supplementary Table S3).

J1a1a1a1a1a-Z2324 branch splits into J1a1a1a1a1a1-PF4881 and J1a1a1a1a1a2-ZS2524 (Figure2, Supplementary Figure S1). J1a1a1a1a1a2-ZS2524 coalesces ~5.7 kya (95% HPD = 4.4-7.0 kya) and occurs in Armenians, Sardinians, and Egyptians (Supplementary File S1, Supplementary Table S4). J1a1a1a1a1a1-PF4881 branch coalesces ~5.7 kya (95% HPD = 4.5-7.0 kya). Six Bronze Age and Iron Age samples (3.0-3.9 kya) from the northern and southern Levant fall within this branch^10,16^. The two ancient northern Levantine populations – Alalakh and Ebla – bear substantial autosomal DNA ancestry from the southern Levant^10^ (Supplementary Table S3).

J1a1a1a1a1a1-PF4881 splits into three branches (Supplementary Figure S1). One Middle Bronze Age individual (3.7 kya) from the Alalakh population belongs to J1a1a1a1a1a1a-Z2329 branch^10^ (Supplementary Table S3). The next branch – J1a1a1a1a1a1b-B2069 – is a minor one and contains one Armenian and one Albanian individuals (Supplementary Table S4). J1a1a1a1a1a1c-B2070 branch is a minor one as well. It is represented with one Jordanian individual and two individuals from the Americas, also one Middle Bronze Age individual from the southern Levant (3.8 kya)^16^ (Supplementary Table S3).

J1a1a1a1a1a1a-Z2329 branch splits into three branches (Supplementary Figure S1). J1a1a1a1a1a1a2-B877 is specific to Jewish Cohens. The four apparently non-Jewish individuals are from Canada, Peru, and the USA: the countries where Jewish communities are known^21^, and the Jewish ancestry was revealed among the native populations^4^. Three Jewish individuals whose Cohen status is uncertain are from the Ashkenazi and Iraqi communities. Interestingly, their lineages coalesce to the same node as all the Cohen lineages. The TMRCA of this branch overlaps with the previous estimate^13^ and equals to ~3.2 kya (95% HPD = 2.4-4.0 kya) (Table 1, Supplementary File S1), which is older than the TMRCA of J1a1a1b1-L817, the other Jewish lineage in haplogroup J1-M267. Intriguingly, an ancient member of J1a1a1a1a1a1a2-B877 branch is found again in Rome, this time in the Imperial period (27 BCE – 300 CE), which is characterized by an increase in the eastern Mediterranean ancestry^14^. Among the nine sequenced positions, two have derived alleles and seven have ancestral alleles, hence this can be from the sister branch of the current Jewish one. J1a1a1a1a1a1a3-Y12510 is a minor branch of J1a1a1a1a1a1a-Z2329 and is represented by one Yemeni and two Qatari individuals. Its TMRCA is 5.1 kya (95% HPD = 3.9-6.4 kya) (Supplementary File S1). One likely member of this branch is found in the Middle Bronze Age (3.5 kya) Levant^10^ (Supplementary Table S3).

J1a1a1a1a1a1a1-L858 branch – coalescing ~5.3 kya (95% HPD = 4.1-6.5 kya) – splits intensively yielding a large number of downstream branches (Figure 2, Supplementary Figure S1, Supplementary File S1). Many branches exclusively include members of the Arabic-speaking population. In contrast, only a small number of minor branches contains only non-Arabic speaking population members. These branches bear fewer than four individuals per branch. Other non-Arabic speaking individuals are scattered as singleton lineages in the Arabic-specific branches. Samples of two different Arabic populations coalesce mostly between ~2 to ~5 kya as many population-specific branches do (Supplementary Figure S1, Supplementary File S1). It is worth to stress that more than half (~56%) of the members of the modern haplogroup J1-M267 belongs to this 5.3 ky old J1a1a1a1a1a1a1-L858 branch. The proportion becomes even larger (~88%) considering haplogroup J1a1a1-P58 chromosomes only. The great majority (~80%) of these is distributed in the Arabian Peninsula, the Levant, southern Mesopotamia, and Egypt. Another ~4% are dispersed in East Africa among the Afro-Asiatic speaking populations of Ethiopia. The ancient J1a1a1a1a1a1a1-L858 samples are found mostly in the southern Levantine Middle Bronze Age (3.4 kya) assemblages^16^. Other members are found in Achaemenid period Lebanon (2.5 kya)^7^ and Late Antiquity Rome (1.5 kya)^14^ (Supplementary Table S3).

**References**

1. Jones, E. R. *et al.* Upper Palaeolithic genomes reveal deep roots of modern Eurasians. *Nat. Commun.* **6**, 8912 (2015).

2. Narasimhan, V. M. *et al.* The formation of human populations in South and Central Asia. *Science* **365**, (2019).

3. Şeker, N. Forced population movements in the Ottoman Empire and the early Turkish republic: an attempt at reassessment through demographic engineering. *Eur. J. Turk. Stud. Soc. Sci. Contemp. Turk.* (2013).

4. Ongaro, L. *et al.* The genomic impact of European colonization of the Americas. *Curr. Biol.* S0960982219313065 (2019) doi:10.1016/j.cub.2019.09.076.

5. Cinnioğlu, C. *et al.* Excavating Y-chromosome haplotype strata in Anatolia. *Hum. Genet.* **114**, 127–148 (2004).

6. Yunusbayev, B. *et al.* The Caucasus as an asymmetric semipermeable barrier to ancient human migrations. *Mol. Biol. Evol.* **29**, 359–365 (2012).

7. Haber, M. *et al.* A genetic history of the Near East from an aDNA time course sampling eight points in the past 4,000 years. *Am. J. Hum. Genet.* (2020) doi:10.1016/j.ajhg.2020.05.008.

8. Wang, C.-C. *et al.* Ancient human genome-wide data from a 3000-year interval in the Caucasus corresponds with eco-geographic regions. *Nat. Commun.* **10**, 1–13 (2019).

9. Lazaridis, I. *et al.* Genetic origins of the Minoans and Mycenaeans. *Nature* **548**, 214–218 (2017).

10. Skourtanioti, E. *et al.* Genomic history of Neolithic to Bronze Age Anatolia, northern Levant, and southern Caucasus. *Cell* **181**, 1158-1175.e28 (2020).

11. Järve, M. *et al.* Shifts in the genetic landscape of the western Eurasian steppe associated with the beginning and end of the Scythian dominance. *Curr. Biol.* **29**, 2430-2441.e10 (2019).

12. Haber, M. *et al.* Insight into the genomic history of the Near East from whole-genome sequences and genotypes of Yemenis. *bioRxiv* 749341 (2019) doi:10.1101/749341.

13. Behar, D. M. *et al.* The genetic variation in the R1a clade among the Ashkenazi Levites’ Y chromosome. *Sci. Rep.* **7**, 14969 (2017).

14. Antonio, M. L. *et al.* Ancient Rome: A genetic crossroads of Europe and the Mediterranean. *Science* **366**, 708–714 (2019).

15. Haber, M. *et al.* Continuity and admixture in the last five millennia of Levantine history from ancient Canaanite and present-day Lebanese genome sequences. *Am. J. Hum. Genet.* **101**, 274–282 (2017).

16. Agranat-Tamir, L. *et al.* The genomic history of the Bronze Age southern Levant. *Cell* **181**, 1146-1157.e11 (2020).

17. Feldman, M. *et al.* Ancient DNA sheds light on the genetic origins of early Iron Age Philistines. *Sci. Adv.* **5**, eaax0061 (2019).

18. Schuenemann, V. J. *et al.* Ancient Egyptian mummy genomes suggest an increase of Sub-Saharan African ancestry in post-Roman periods. *Nat. Commun.* **8**, 1–11 (2017).

19. Marcus, J. H. *et al.* Genetic history from the Middle Neolithic to present on the Mediterranean island of Sardinia. *Nat. Commun.* **11**, 1–14 (2020).

20. Lazaridis, I. *et al.* Genomic insights into the origin of farming in the ancient Near East. *Nature* **536**, 419–424 (2016).

21. Johnson, P. *A History of the Jews*. (HarperCollins Publishers, 1988).
