# Supplementary material for: Origin and diffusion of human Y chromosome haplogroup J1-M267
Source: Sci Rep. 2021 Mar 23;11:6659. doi: 10.1038/s41598-021-85883-2 (PMC7987999; doi:10.1038/s41598-021-85883-2)
Supplement: Supplementary file 7 — Supplementary File S1 Legend. [file 41598_2021_85883_MOESM7_ESM.docx]

Supplementary File S1. Haplogroup J1-M267 Bayesian MCC Tree.

Maximum clade credibility (MCC) tree of haplogroup J1-M267 Bayesian phylogenetic analysis. In the analysis one member of haplogroup J2-M172 is used for the proper rooting. The phylogeny, medians and 95% HPD intervals of posterior probabilities of node heights and other parameters can be viewed in “FigTree” program (<http://tree.bio.ed.ac.uk/software/figtree>).
