# Supplementary material for: Origin and diffusion of human Y chromosome haplogroup J1-M267
Source: Sci Rep. 2021 Mar 23;11:6659. doi: 10.1038/s41598-021-85883-2 (PMC7987999; doi:10.1038/s41598-021-85883-2)

Supplementary Figure S1. Haplogroup J1−M267 Maximum Likelihood Tree

Denoted on the edges of the tree are the names of the defining markers with which we call the branches and the overall numbers of markers defining the branches. The marker names you can find in the Supplementary Table S2.

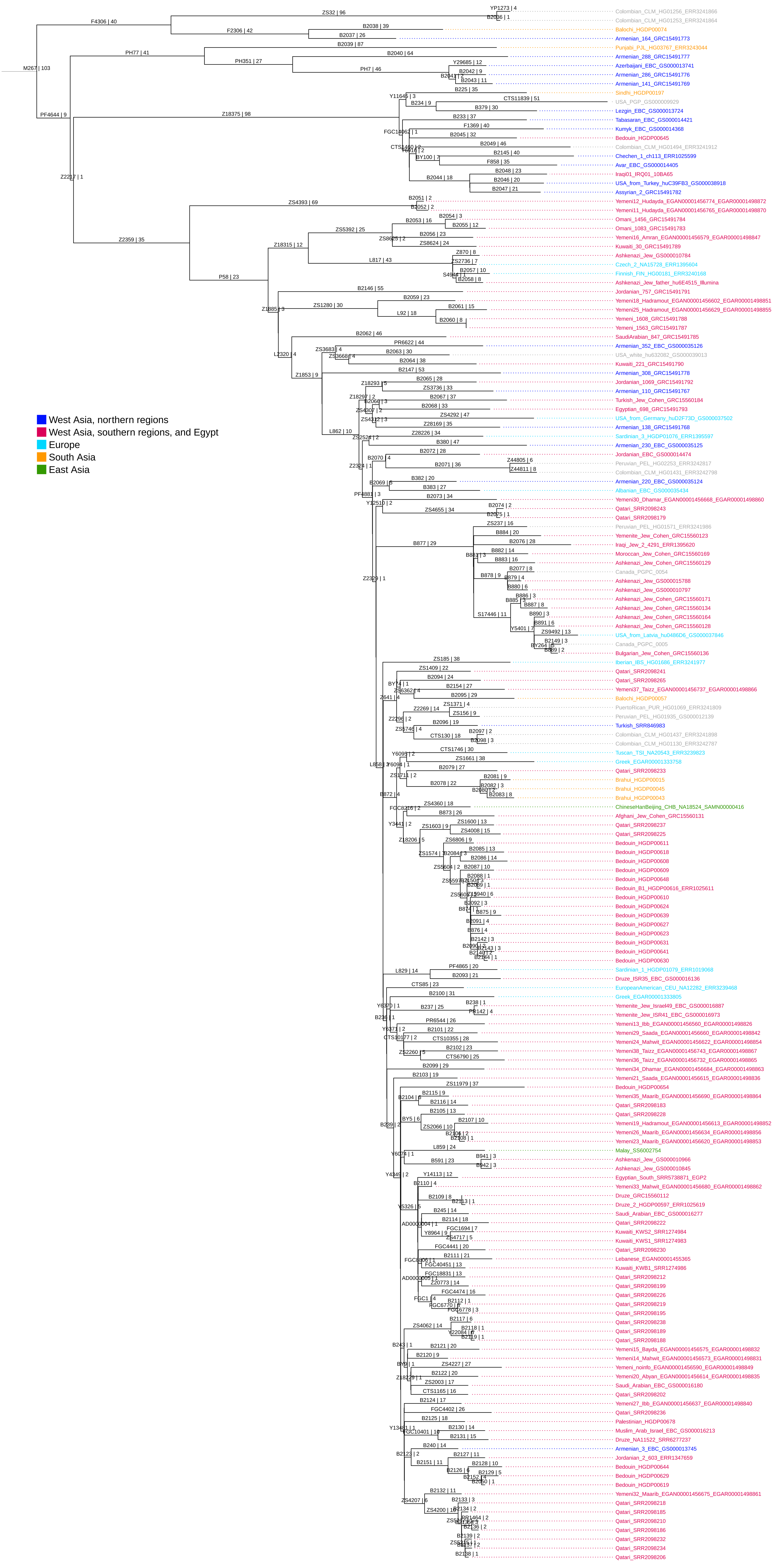

Supplement: Supplementary file 1 — Supplementary Figure S1. [file 41598_2021_85883_MOESM1_ESM.pdf]
