# Supplementary material for: Origin and diffusion of human Y chromosome haplogroup J1-M267
Source: Sci Rep. 2021 Mar 23;11:6659. doi: 10.1038/s41598-021-85883-2 (PMC7987999; doi:10.1038/s41598-021-85883-2)

Supplementary Figure S2. Ancient individuals belonging to haplogroup J1−M267

Colors mark the antiquity. Detailed information about the ancient individuals are provided in Supplementary Table S3. The map was generated in RStudio v1.2.5019 (RStudio Team. RStudio: Integrated Development Environment for R. RStudio, Inc., 2019) on the basis of R v3.6.1 (R CoreTeam. R: A language and environment for statistical computing. R Foundation for Statistical Computing, 2019). The base map was downloaded from http://tapiquen−sig.jimdo.com.

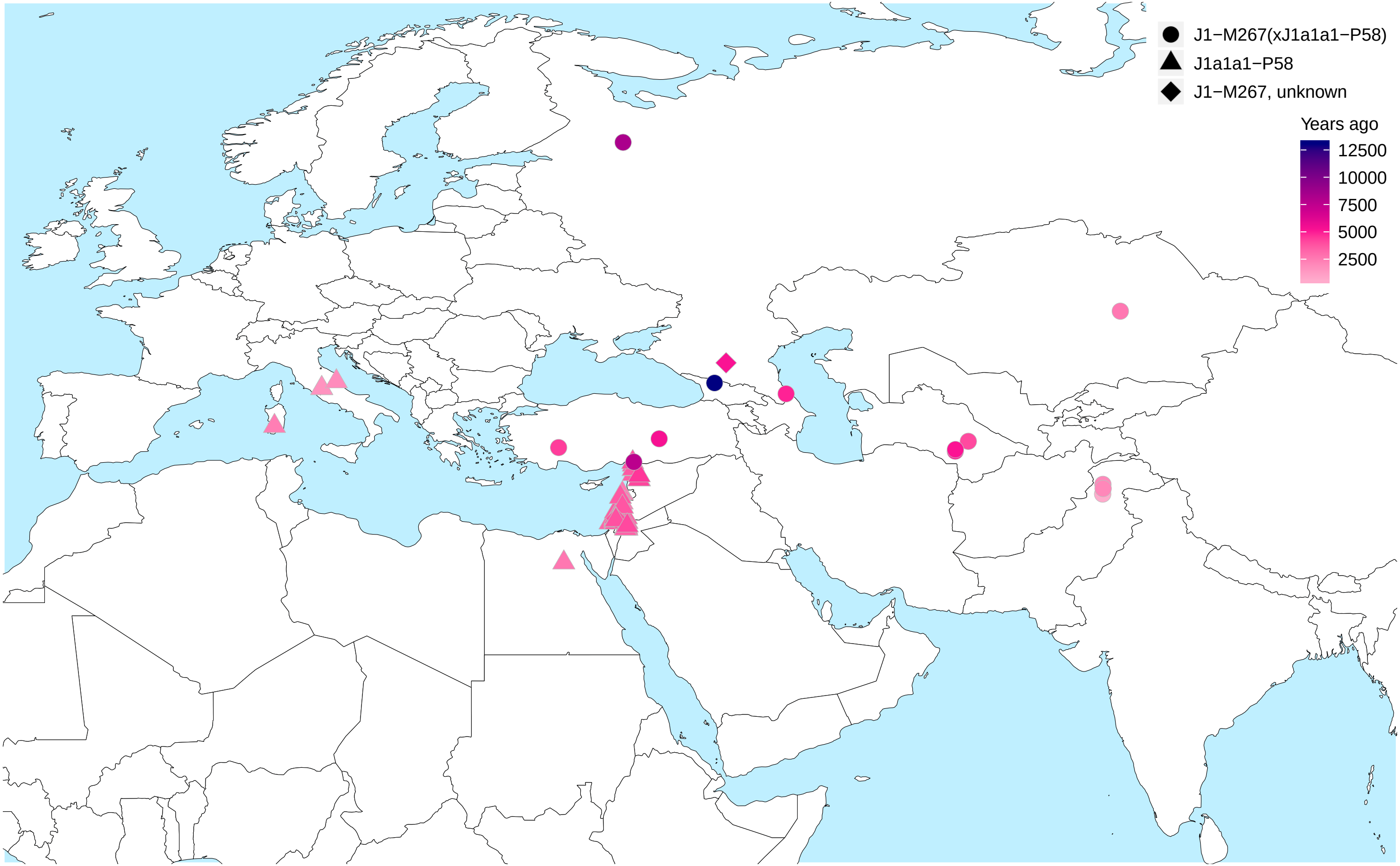

Supplement: Supplementary file 2 — Supplementary Figure S2. [file 41598_2021_85883_MOESM2_ESM.pdf]
